# Supplementary material for: Comparison of Structural Features of CRISPR-Cas Systems in Thermophilic Bacteria
Source: Microorganisms. 2023 Sep 10;11(9):2275. doi: 10.3390/microorganisms11092275 (PMC10536717; doi:10.3390/microorganisms11092275)

Figure S1. RNA secondary structures and minimum free energy of all repeat sequence.

1.ACCTTTCAATTATTTCTGAGTTGCATC -0.20 kcal / mol Thermofilum adornatus

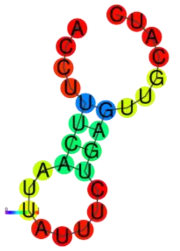

2.ACTATTTTCAGGATAGGTAGGCTAAAAAC -2.40 kcal / mol Thermoanaerobacter wiegelii Rt8.B1 *and Thermoanaerobacter italicus* Ab9

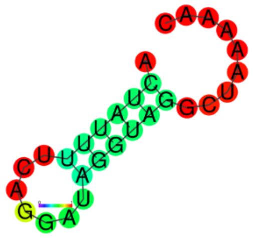

3.AGGTTTGTTGCTTCATGTGTGTAAGAAAA -2.90 kcal / mol Thermovibrio ammonificans HB-1

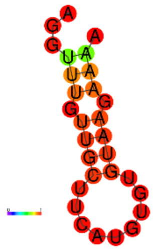

4.AGTAGTCCCCACGCACGTGGGGATGGCCCG -15.50 kcal/mol Thermus scotoductus SA-01

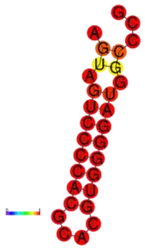

5. AGTCCGAAAGAAGGTCCTCTCTGAAAGAGATGG -5.78 kcal/mol Pseudothermotoga elfii DSM 9442 = NBRC 107921

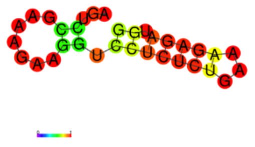

6. AGTTTTTACGATACCTATGAGGAATTGAAACA -3.90 kcal / mol Thermodesulfobium narugense DSM 14796

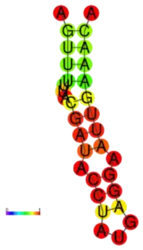

7. ATCAATCAATTACTTATACATCTAA 0.00 kcal/mol Thermocrinis albus DSM 14484

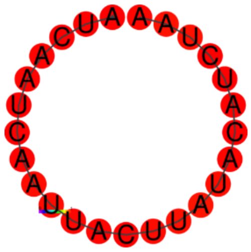

8. ATCATCTGACTACCTGACTACCG 0.00kcal/mol Thermus aquaticus Y51MC23

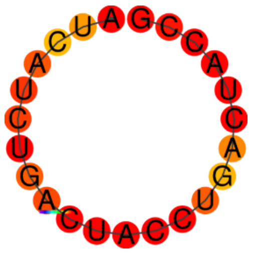

9. ATGGCCCGGAAACGCGCGGGGCG -9.20 kcal / mol Thermobispora bispora DSM 4383
3

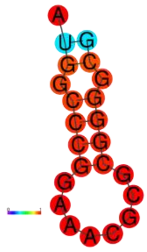

*10. ATTGTCCCCACACGCGTGGGGGTGTACACCG -11.70 kcal / mol Thermobaculum terre
num* ATCC BAA-798

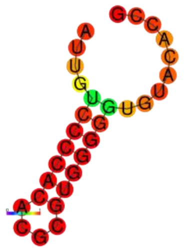

11.ATTTACATCCCTCATAGTTCAGATAAAAC 0.00 kcal/mol Thermoanaerobacter mathranii su
bsp. mathranii str. A3

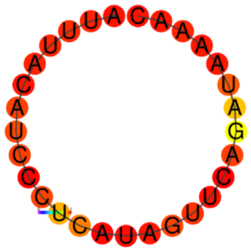

12. ATTTCAATTCCTACAAGGTAAGGTACAAAC -1.10 kcal / mol Thermosipho africanus TC F52B

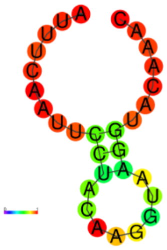

13. ATTTCAATTCCTCCAAGGTAAGGTAAAAAC -0.80 kcal / mol Thermosipho melanesiensis BI429 and Thermosipho melanesiensis strain 431

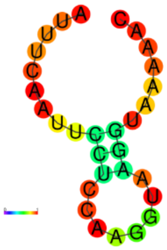

14. ATTTCAATTCCTGCAAGGTAAGGTACAAAC -2.10 kcal / mol Thermosipho africanus T CF52B

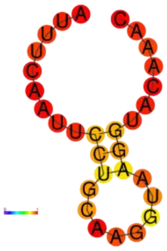

15. ATTTCTATTCCTCATAGGTAGATTCTAAAC 2.30 kcal / mol Thermosipho melanesiensis B I429 and Thermosipho melanesiensis strain 431

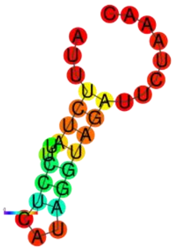

16. CAACTCCACACGGTACATTAGAAAC 0.00 kcal / mol Thermocrinis albus DSM 14484

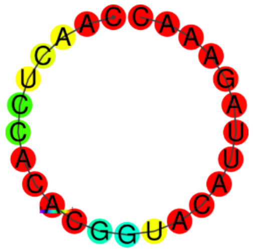

17.CACTATTTTCAGGATAGGTAGGCTAAAAAC -2.40 kcal / mol Thermoanaerobacter italicus Ab9

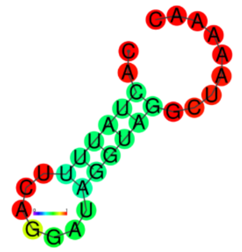

18. CAGTTCTAGCAGTTAGAACGAGTTGTAAACCT -5.10 kcal/mol Thermoanaerobacter kivu i strain DSM 2030

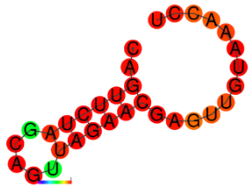

19. CCAGGGCCGCCTGGGCGAGGAGGA -8.70 kcal/mol Thermus thermophilus SG0.5JP17-16

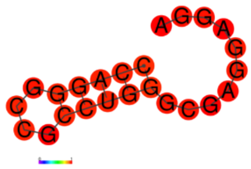

20. CCATCTCCGCCTTGAGCTCCTGGCGCA -5.40 kcal/mol Thermus thermophilus SG0.5JP 17-16

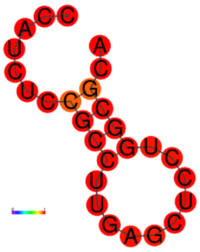

21. CCCTTTTCACCATAAAATCAATACTTTTCAAC 0.00 kcal/mol Thermoanaerobacterium sac charolyticum JW_SL-YS485

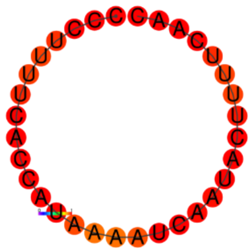

22. CCGTTCCCCGCGGGCCGGAAGGGGTG -7.70 kcal/mol Thermomonospora curvata DS M 43183

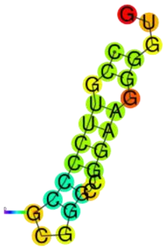

23. CCTTCAATTCTTTCTTAGTTGCATC 0.00 kcal/mol Thermofilum adornatus

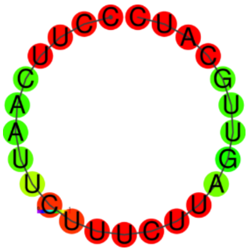

24. CGACATCCCGCGCTGACCGGGGGCCACGG -7.20 kcal/mol Thermobispora bispora DSM 43833

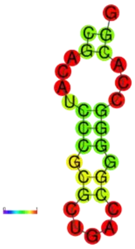

25. CGACCGGCGGCCCGCTCACGGCC -5.40 kcal/mol Thermobispora bispora DSM 43833

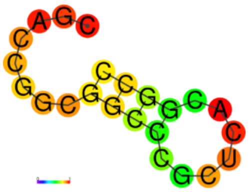

26. CGCCGTCGTTTTTATAAGGGGAATAGAAACCC -3.90 kcal/mol Thermoanaerobacter kivui strain DSM 2030

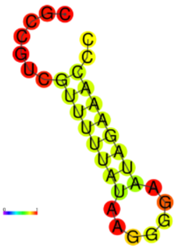

27. CGCGGGCCCCGCGCATGCGGGACGCGGGC -15.50 kcal/mol Thermobispora bispora DSM 43833

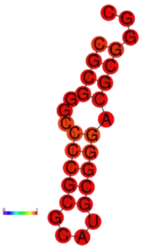

28. CGGACCACCCCCGCCTGCGCGGGGAGCAC -12.70 kcal/mol Thermomonospora curvata DSM 43183

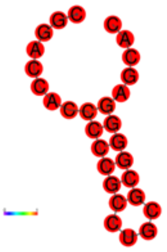

29. CGGCCCCGCATCGCCCGGGCGCCGC -12.70 kcal/mol Thermobispora bispora DSM 43833

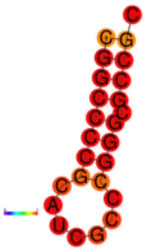

30. CGGCGGCGCGCGGACACGACGGGTGA -6.30 kcal/mol Thermobispora bispora DSM 43833

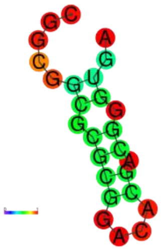

31. CGGGCCATCCCCACGCGTGTGGGGACTAC -12.60 kcal/mol Thermus scotoductus SA-01

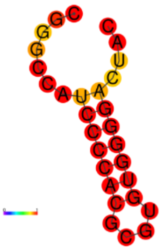

32. CGGGTTTCAATACTTCCTTAGAGGTATGGAAAC -10.30 kcal/mol Thermotoga neapolitana DSM 4359

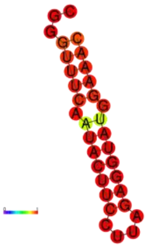

33. CGGTCCATCCCCACGGGCGTGGGGACTAC -12.60 kcal/mol Thermus oshimai JL-2

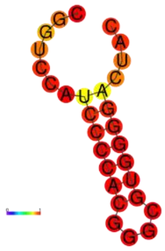

34. CGGTTCACCCCCACGCGTGTGGGGACAAT -11.60 kcal/mol Thermobaculum terrenum ATCC BAA-798

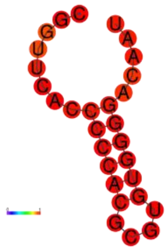

35. CGTAGTCCCCACACGCGTGGGGATGGACC -14.60 kcal/mol Thermus sp. CCB_US3_UF1

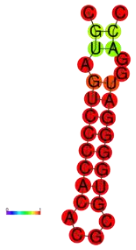

36. CGTGCAGGCCTCGTATCTGCTCTGAGGTGCAAG -8.10 kcal/mol Thermobaculum terrenum ATCC BAA-798

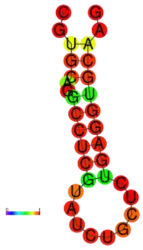

37. CGTTCACCACCAGCAGGGCCGCC -0.80 kcal/mol Thermomonospora curvata DSM 43183

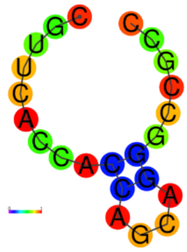

*38. CTAAAACTGAAGAAGTGTTAGAGGA -3.60 kcal/mol Thermocrinis ruber s*train DSM 23557

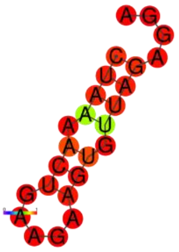

39. CTATAGTTGTTGGTTGTTGGTTTT 0.00 kcal/mol Thermobaculum terrenum ATCC BAA-798

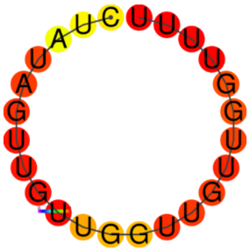

40.CTCCCGTCCCCGGGCCCAGCGGGC -7.20 kcal/mol Thermomonospora curvata DSM 43 183

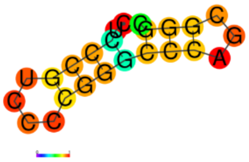

41. CTCGCAGTGACAAATGTGATCAGGG -4.40 kcal/mol Thermodesulfatator indicus DSM 15286

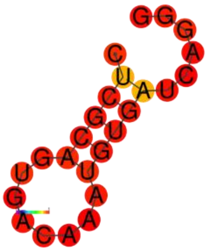

42. CTGCTCCCCGCGCACGCGGGGATGGTCCC -14.30 kcal/mol Thermomonospora curvata DSM 43183

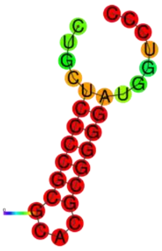

43. CTGTAACGCGGATACCGCGTTACA -14.40 kcal/mol Thermobacillus composti KWC4

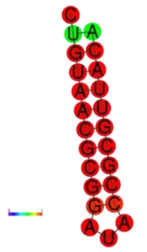

44. CTTCTAAACCTACATAGGATATTTCAAAC -1.30 kcal/mol Thermoanaerobacterium M0795

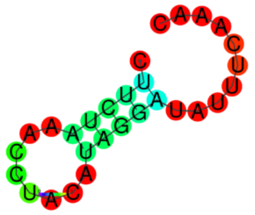

45. CTTTATATCCCACATGGTTCAGATGAAAC -0.60 kcal/mol Thermodesulfovibrio yellowstonii DSM 11347

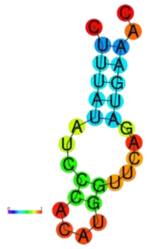

46. CTTTATATCTCACATGGTTCAGATGAAAC -0.40 kcal/mol Thermodesulfobacterium commune DSM 2178

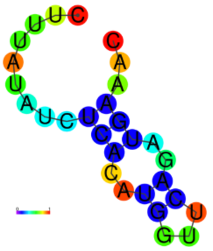

47. CTTTCAACTCCACACGGTACATTAGAAAC 0.00 kcal/mol Thermocrinis albus DSM 14484

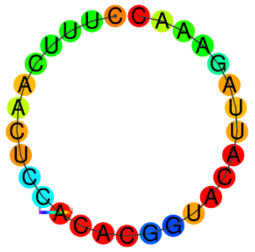

48. CTTTCAACTCCACACGGTACATTAGGAAC -0.90 kcal/mol Thermocrinis ruber strain DSM 23557

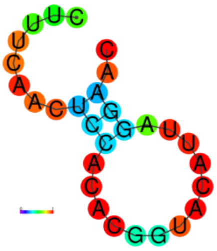

49. CTTTCAATTCATTCGTTTCTGATAC 0.00 kcal/mol Thermofilum adornatus

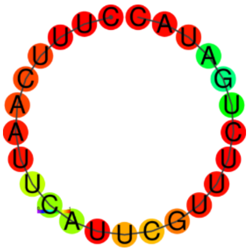

50.CTTTCAATTCATTCTTTTCTGATAC 0.00 kcal/mol Thermofilum adornatus

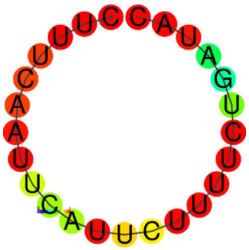

51. CTTTCAATTCCAGTATGGTTGGATTAAATC -3.10 kcal/mol Thermoanaerobacter kivui strain DSM 2030

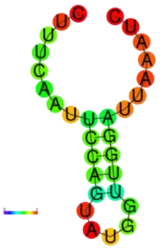

52. CTTTCAATTCCGTTCTACGGAATTTGGTCTTGAGGC -7.70 kcal/mol Thermotoga caldifontis AZM44c09

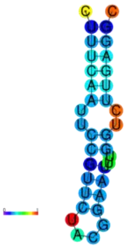

53. CTTTCAATTCCTTATAGGTAGGCTAAAAAC -0.80 kcal/mol Thermoanaerobacter wiegelii Rt8.B1

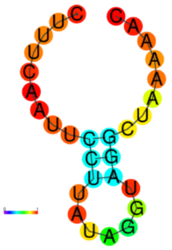

54. *CTTTCAATTCTTCTAAAGTCTTATTGGAAC -2.00 kcal/mol Thermococcus litoralis* DSM 5473

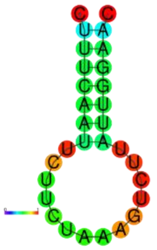

55. *CTTTCAATTCTTTCTGAGTTGCATC -4.30 kcal/mol Thermofilum adornatus*

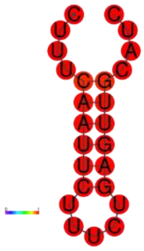

56. *CTTTCAATTCTTTCTTAGTTGCATC 0.00 kcal/mol Thermofilum adornatus*

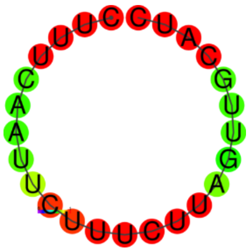

57. *CTTTCAATTCTTTTAAAGTCTTATTGGAAC -2.00 kcal/mol Thermococcus litoralis* DSM 5473

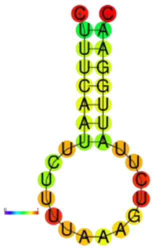

58. *CTTTGAACCGTACCTATAAGGGTTTGAAAC -5.90 kcal/mol Thermus* sp. CCB_US3_UF1

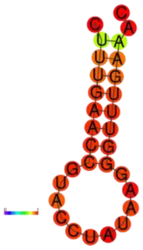

59. *CTTTGAATTCTTTCTGAGTTGCATC -2.70 kcal/mol Thermofilum adornatus*

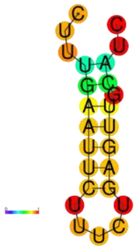

60. CTTTTGACCGTACCTATGAGGGTTTGAAAC -3.80 kcal/mol Thermus aquaticus Y51MC2
3

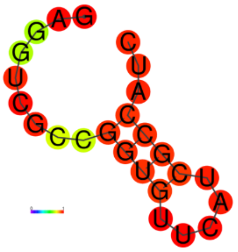

*61. GAGGTCGCCGGTGTTCATCGCCATC -4.60 kcal/mol Thermobispora bispora DSM 4383
3*

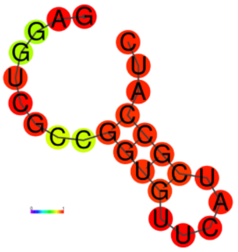

62. GATGCAACTGAGAAAGAATTGAAAGC 0.00 kcal/mol Thermobispora bispora DSM 438
33

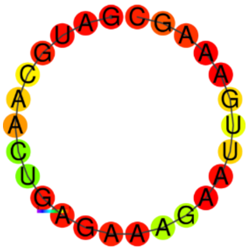

63. GATTCAATCGAACCGATACGGAATGGAAAC -2.60 kcal/mol Thermovirga lienii DSM 17
291

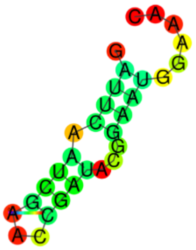

64. GATTTAAGCGGGCGCATCCGTTTTGCGGA -5.80 kcal/mol Thermobacillus composti K
WC4

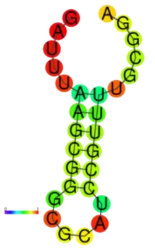

65. GCCACCGGGCCTCAGCGCCACCGTC -3.80 kcal/mol Thermobispora bispora DSM 43
833

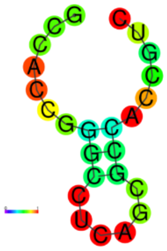

66. GCGAGGGAGGCTGCAGCGTGCAT -3.00 kcal/mol Thermobacillus composti KWC4

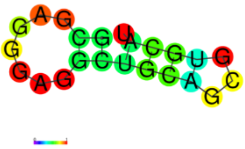

67. GCGCCCCAGGAGCACGGTGCGCGG -9.60 kcal/mol Thermobispora bispora DSM 438
33

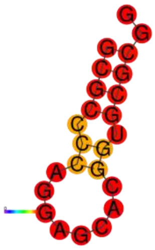

68. GCGGAGCTTCGGGCCCCGTGGGA -7.70 kcal/mol Thermobispora bispora DSM 43833

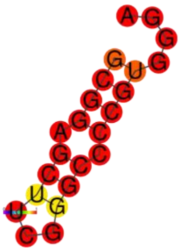

69. GCGGCGGCAGGCGCGTGAGGGGCCGCG -10.60 kcal/mol Thermomonospora curvata
DSM 43183

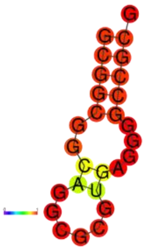

70. GCGGGACCCCGGACGGGCGGGCGG -6.50 kcal/mol Thermobispora bispora DSM 43
833

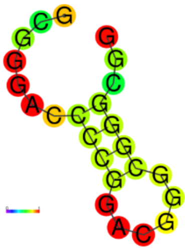

71. GCGTTCTCCTGGCCCGGCAGGGCCGG -13.80 kcal/mol Thermomonospora curvata DSM 43183

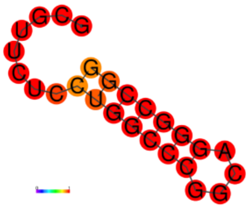

72. GGAGATGAATGCAAGGTTTGAGGCT 0.00 kcal/mol Thermodesulfobacterium geofontis OPF15

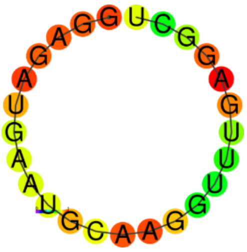

73. GGAGGAGTCGAACCTCCGACCTC -7.90 kcal/mol Thermocrinis ruber strain DSM 23557

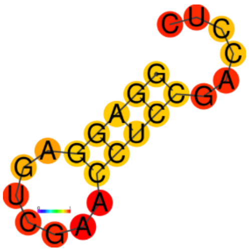

74. GGATCATCCCCGCATGCGCGGGGAGCAC -14.30 kcal/mol Thermocrinis ruber strain DSM 23557

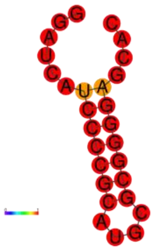

75. GGGACCATCCCCGCGTGCGCGGGGAGCAG -14.30 kcal/mol Thermomonospora curvata DSM 43183

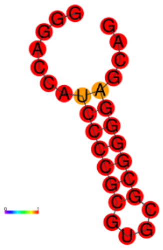

76. GGGATCATCCCCGCGTGCGCGGGGAGCAC -14.30 kcal/mol Thermobispora bispora DSM 43833

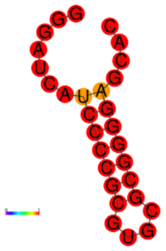

*77. GGGATCATCCCCGCGTGCGCGGGGAGCAG -14.38 kcal/mol Thermomonospora curvat
a* DSM 43183

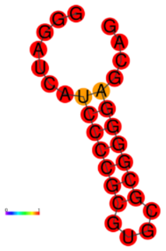

*78. GGTTGCGATCCCTCTGAGGGGTGATGAGGGC -12.60 kcal/mol Thermomonospora curv
ata* DSM 43183

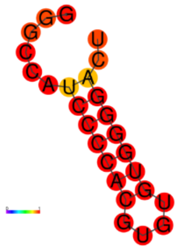

*79. GGTTGCGATCCCTCTGAGGGGTGATGAGGGC -7.10 kcal/mol Thermomonospora curvat
a* DSM 43183

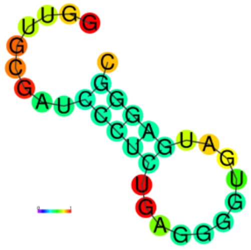

80. GGTTTCAATACTTCCTTTGAGGTATGGAAAC -10.10 kcal/mol Thermotoga sp. RQ7

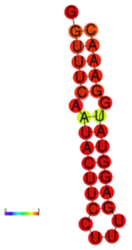

81. GGTTTTTTCAATTCGAATAATCGAAAGAAC -4.30 kcal/mol Thermotoga petrophila T RK
U-1

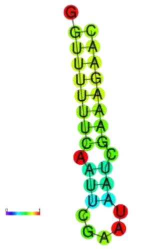

82. GTAGAAACCTGCCCTACTTCAAAAGGGATTGCGAC -5.00 kcal/mol Thermodesulfobacterium commune DSM 2178

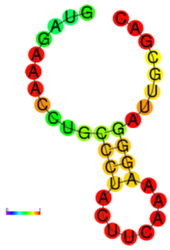

83. GTAGGAAACAAGACCTCATTTAAAAGGGATTGCGAC -5.30 kcal/mol Thermodesulfovibrio yellowstonii DSM 11347

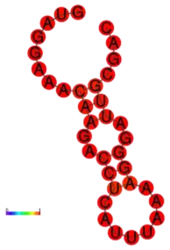

84. GTAGTCCCCACACGCGTGGGGATGGACCG -14.30 kcal/mol Thermus oshimai JL-2

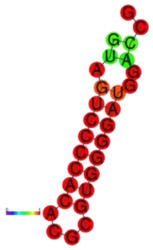

85. GTAGTCCCCACGCACGTGGGGATGGACC 14.30 kcal/mol Thermus sp. CCB_US3_UF1

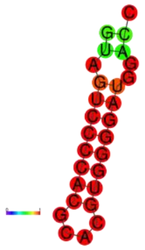

86. GTAGTCCCCACGCATGTGGGGATGGCCCG -15.48 kcal/mol Thermus scotoductus SA-01

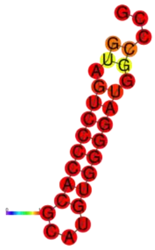

87. GTCAGTGCGGGCGGGGGAGCAGG -1.70 kcal/mol Thermobispora bispora DSM 43833

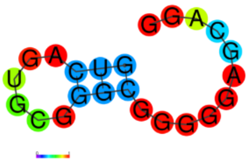

88. GTCCGGGAGGCGCGGATGAGCGCC -9.60 kcal/mol Thermobispora bispora DSM 438
33

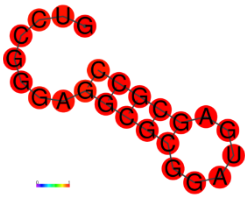

89. GTCCTCATCACCCCTCAGAGGGATCGCAAC -4.50 kcal/mol Thermomonospora curvata
DSM 43183

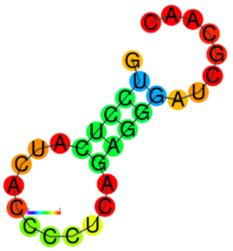

90. GTCCTCATCACCCCTCGGAGGGATCGCAAC -4.40 kcal/mol Thermomonospora curvata
DSM 43183

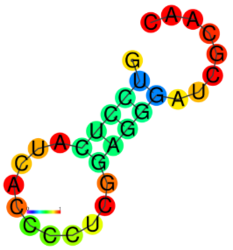

*91. GTCGCAATCCCCTGACGGGGAAGCATCTCGTGCAAC -10.90 kcal/mol Thermus oshima
i* JL-2

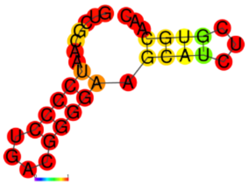

92. GTCGCAATCCCCTTACGGGGAAGCCACTTTTGCAAC -10.70 kcal/mol Thermus sp. CC
B_US3_UF1

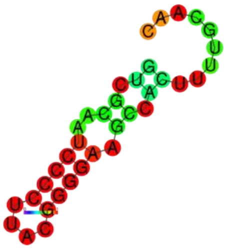

93. GTCGCAATCCCCTTACGGGGCTAAGTGG -8.40 kcal/mol Thermus aquaticus Y51MC23

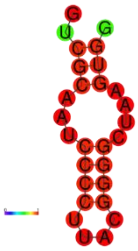

94. GTCGCAATCCCCTTACGGGGCTAAGTGGTTTGCAAC -8.90 kcal/mol Thermus aquaticus Y51MC23

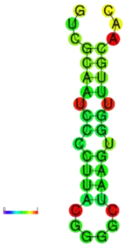

95. GTCGCAATCCCCTTACGGGGCTCAATCCCTTGCAAC -7.40 kcal/mol Thermus thermophilus HB27

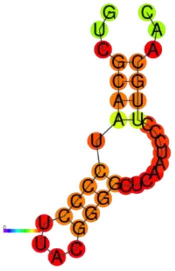

96. GTCGCAATCCCGTCTACTTTTTCGGGCATTTCAAC -4.80 kcal/mol Thermovibrio ammonificans HB-1

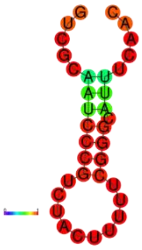

97. GTCGCAATCCCTTATTCTTCAGGGAATTTTTCTAAC -6.00 kcal/mol Thermodesulfobium narugense DSM 14796

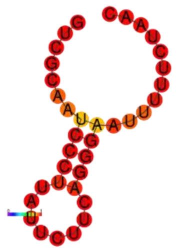

98. GTCGCCCGCTGTGCGCGGGCGTGGGTTGAAAC -14.60 kcal/mol Thermobacillus comp osti KWC4

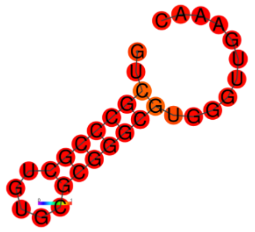

99. GTCGCTCATCACCCCTGGAGGGATCGCAAC -5.00 kcal/mol Thermomonospora curvata DSM 43183

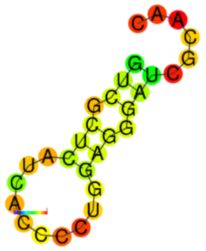

100. GTGAGAAAACCTTGCCTGATTAAGAAGGCATTACGAC -7.40 kcal/mol Thermodesulfatat or indicus DSM 15286

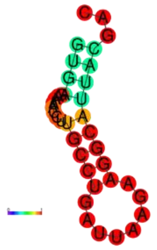

101. GTGCTCAACGCCTTACGGCATCAGAGGTAGAGGCAC -9.00 kcal/mol Thermosynechoc occus sp. NK55

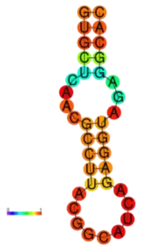

102. GTGCTCCCCGCGCACGCGGGGATGATCCC -14.30 kcal/mol Thermobispora bispora DSM 43833

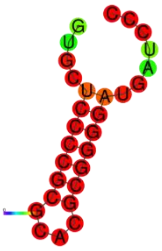

103. GTGCTCCCCGCGCAGGCGGGGGTGATCCG -14.80 kcal/mol Thermomonospora curva ta DSM 43183

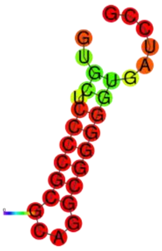

104. GTTAAAAAACCTAATTCCATAAATGGAATTCAAAC -5.20 kcal/mol Thermosipho sp. 106 3

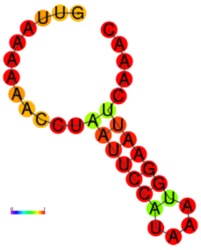

105. GTTACTAGCTTACCTATGAGGGGTTGAAAC -2.90 kcal/mol Thermosediminibacter ocea ni DSM 16646

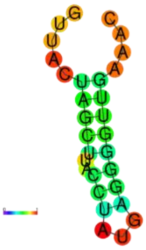

106. GTTACTAGCTTACCTATGAGGGGTTGAAACAT -3.50 kcal/mol Thermosediminibacter oc eani DSM 16646

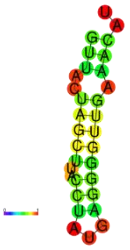

107. GTTCACAGCCTAACTAAAAGGAATGGAAAC -3.50 kcal/mol Thermodesulfatator indicus DSM 15286

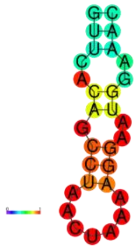

108. GTTCATATTCCTCTTAGGAAGATAAAAAC -3.10 kcal/mol Thermotoga petrophila T RK U-1

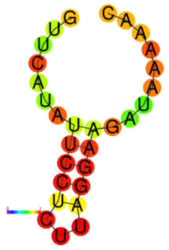

109. GTTCCAATAAGACTTTAAAAGAATTGAAAG 0.00 kcal/mol Thermococcus litoralis DSM 5473

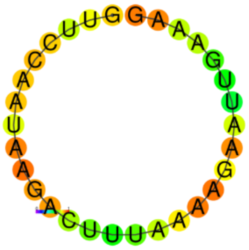

110. GTTCCTAATGTACCGTGTGGAGTTGAAAG -3.70 kcal/mol Thermocrinis ruber strain D SM 23557

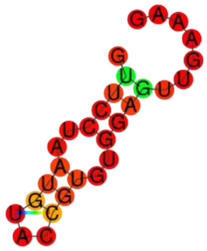

111. GTTCCTAATGTACTGTGTGGAGTTGAAAG -2.30 kcal/mol Thermocrinis ruber strain D SM 23557

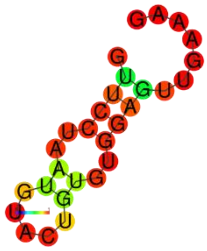

112. GTTCTACTGTAACTTAGAAGTTTTTTGTG -*1.70 kcal/mol Thermoanaerobacter kivui* str ain DSM 2030

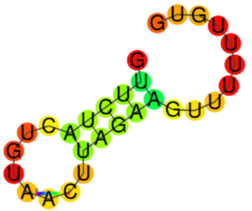

113. GTTCTTAGTCTACCTATAAGGGATTGAAAC -3.90 kcal/mol Thermosediminibacter oceani DSM 16646

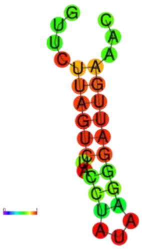

114. GTTGAAAAGTATTGATATTATGTCGAGAAGG -1.90 kcal/mol Thermoanaerobacterium xylanolyticum LX-11

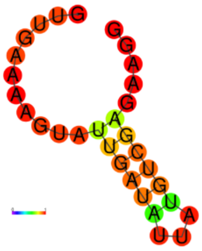

115. GTTGAAACCTGCCCTGGATTAAAAGGGATTGCGAA -6.00 kcal/mol Thermodesulfobacterium geofontis OPF15

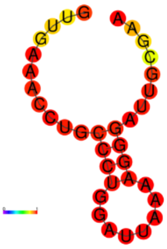

116. GTTGAAATGCCCGAAAAAATCGGCGGGATTGCGAC -8.10 kcal/mol Thermovibrio ammonificans HB-1

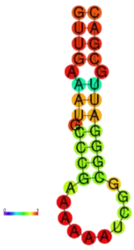

117. GTTGAAATGCCCGAAAAAGTAGACGGGATTGCGAC -7.90 kcal/mol Thermovibrio ammonificans HB-1

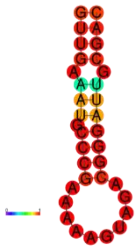

118. GTTGCAAAAGTGGCTTCCCCGCAAGGGGATTGCGAC -14.60 kcal/mol Thermus sp. CCB_US3_UF1

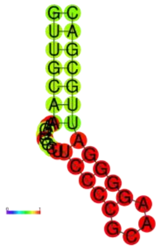

119. GTTGCAAGGGATTGAGCCCCGTAAGGGGATTGCGAC -11.70 kcal/mol Thermus thermophilus HB27 *and Thermus thermophilus* HB8

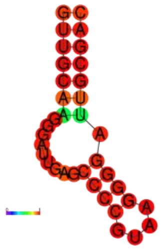

120. GTTGCACGAGATGCTTCCCCGTCAGGGGATTGCGACC -13.30 kcal/mol Thermus oshimai JL-2

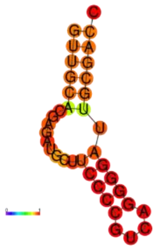

121. GTTGCGATCCCTCCAGGGGTGATGAGCGAC -9.40 kcal/mol Thermomonospora curvata DSM 43183

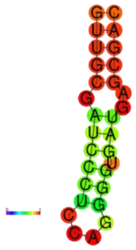

122. GTTGCGATCCCTCTAGGGGTGATGAGCGGAC -7.60 kcal/mol Thermobispora bispora DSM 43833

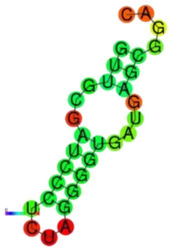

*123. GTTGCGATCCCTCTGAGGGGTGATGAGGAC -6.60 kcal/mol Thermomonospora curvat
a* DSM 43183

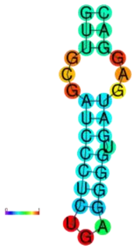

*124. GTTGCGATCCCTCTGAGGGGTGATGAGGACC -7.40 kcal/mol Thermomonospora curv
ata* DSM 43183

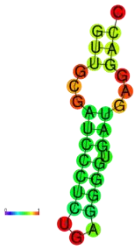

*125. GTTGGAAAACAAGACCTCATTTAAAAGGGATT -2.60 kcal/mol Thermodesulfovibrio yell
owstonii* DSM 11347

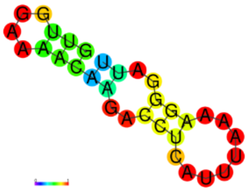

126. GTTTAAATCCCACTTGTTCAAATAAAAC 0.00 kcal/mol Thermovibrio ammonificans HB
-1

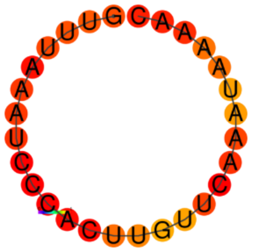

127. GTTTAAATCCCACTTGTTCAGATAAAAC 0.00 kcal/mol Thermovibrio ammonificans HB
-1

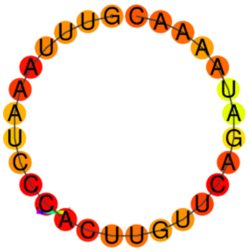

128. GTTTAGAACATACCTATGAGGAATGGAAAC -3.10 kcal/mol Thermosipho sp. 1063

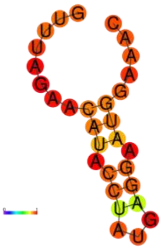

129. GTTTAGAATCTACCTATGAGGAATGAAAAC -0.60 kcal/mol Thermosipho africanus TCF52B

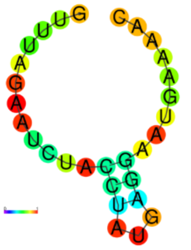

130. GTTTAGAATCTACCTATGAGGAATGGAAAC -2.00 kcal/mol Thermosipho melanesiensis BI429 and Thermosipho melanesiensis strain 431

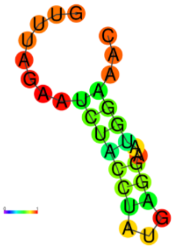

131. GTTTATAACCCACAATGGTTCTACCTAAAC -1.80 kcal / mol

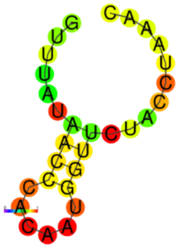

132. GTTTATTAAATGCCTATAGGGGATTGAAAC -3.50 kcal/mol Thermocrinis ruber strain DSM 23557

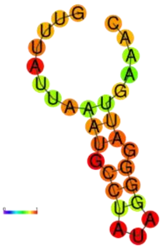

133. GTTTCAAACCCTCATAGGTACGGTCAGAAC -2.00 kcal/mol Thermus sp. CCB_US3_U

F1

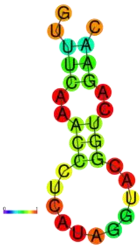

134. GTTTCAATAATTCCTTAGAGGTATGGAAAC -4.10 kcal/mol Thermotoga petrophila TRKU-1*, Thermotoga maritima* strain Tma200, *Thermotoga maritima* strain Tma100*, Thermotoga maritima* MSB8, *Thermotoga naphthophila* RKU-10 *and Thermotoga* sp. RQ2

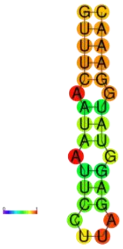

135. GTTTCAATACTTCCTTAGAGGTATGGAAAC -10.30 kcal/mol Thermotoga neapolitana DSM 4359, *Thermotoga maritima* strain Tma200, *Thermotoga maritima* strain Tma100, *Thermotoga maritima* MSB8, *Thermotoga* sp. RQ2, *and Thermotoga* sp. RQ7

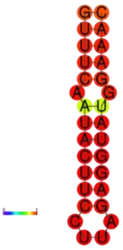

136. GTTTCAATACTTCCTTTGAGGTATGGAAAC -10.10 kcal/mol Thermotoga sp. RQ7 *and Thermotoga neapolitana* DSM 4359

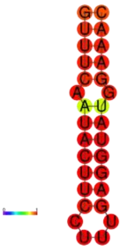

137. GTTTCAATAGTTCCTTAGAGGTATGGAAAC -6.70 kcal/mol Thermotoga petrophila TRKU-1, *Thermotoga naphthophila* RKU-10*, and Thermotoga* sp. RQ2

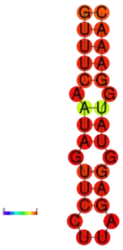

138.GTTTCAATCCACCAAAGAGGAATTTAAAC -2.00 kcal/mol Thermodesulfobacterium geofontis OPF15

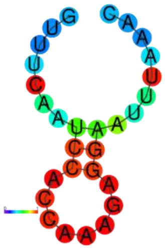

139.GTTTCAATCCCCTATAGGCACTT 0.00 kcal/mol Thermocrinis ruber strain DSM 23557

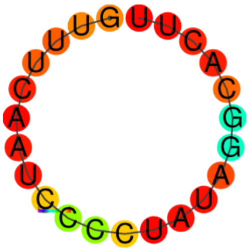

140.GTTTCAATCCCTAATAGGTATGCTAAAAAC -0.50 kcal/mol Thermosipho africanus TCF5 2B

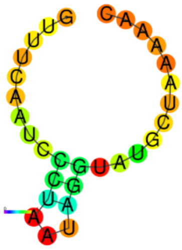

141.GTTTCAATCCCTCTTAGGTAGGCTAAAAAC -1.30 kcal/mol Thermoanaerobacter kivui strain DSM 2030

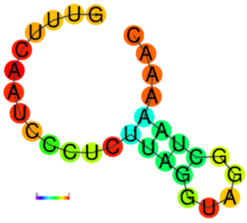

142.GTTTCAATCCCTTATAGGTAAGCTAAAAAC -0.50 kcal/mol Thermoanaerobacter sp. X51 3

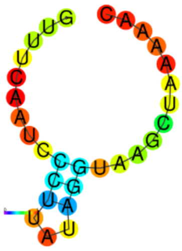

143.GTTTCAATCCTTCCTTAGAGGTATGGAAACA -8.00 kcal/mol Thermotoga sp. RQ7

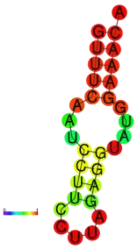

144.GTTTCAATCGAACCTTAGAGGGATGGAAAC -5.00 kcal/mol Thermotoga caldifontis AZ M44c09, *Pseudothermotoga elfii* DSM 9442 = NBRC 107921*, and Thermotoga lettingae* T MO

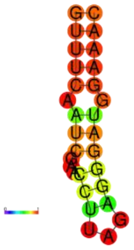

145.GTTTCAATCGAACTTAAGAGGGATGGAAAC -4.40 kcal/mol Thermotoga thermarum DS M 5069

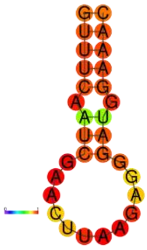

146.GTTTCAATTCCACTATGGTTAGATTAAATC -0.20 kcal/mol Thermoanaerobacterium thermosaccharolyticum DSM 571 *and Thermoanaerobacterium xylanolyticum* LX-11

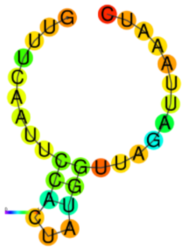

147.GTTTCAATTCCTACAAGGTAAGGTACAAAC -1.20 kcal/mol Thermosipho africanus TCF5 2B

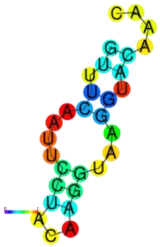

148.GTTTCAATTCCTCATAGGTACGATCAAAAC -0.30 kcal/mol Thermobacillus composti KW C4

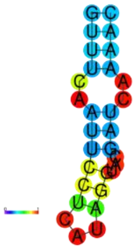

149.GTTTCAATTCCTCATAGGTAGGCTAAAAAC -0.20 kcal/mol Thermoanaerobacter italicus Ab9

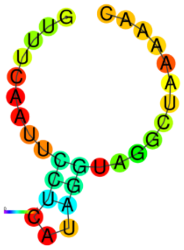

150.GTTTCAATTCCTGATAGGTAGGCTAAAAAC -1.50 kcal/mol Thermoanaerobacterium thermosaccharolyticum DSM 571

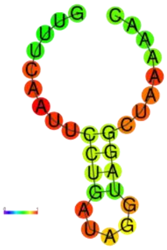

151.GTTTCAATTCCTTATAGGTAGGCTAAAAAC -0.80 kcal/mol Thermoanaerobacterium saccharolyticum JW_SL-YS485, *Thermoanaerobacterium xylanolyticum* LX-11, *Thermoanaerobacter* sp. X513, *Thermoanaerobacter* sp. X514, *Thermoanaerobacter kivui* strain DSM 2030, *Thermoanaerobacter wiegelii* Rt8.B1*, and Thermoanaerobacter mathranii* subsp. mathranii str. A3

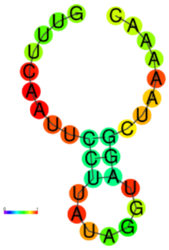

152.GTTTCCATACCTCAAAGGAAGTATTGAAAC -6.80 kcal/mol Thermotoga neapolitana DSM 4359

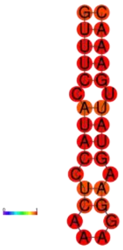

153.GTTTCCATACCTCTAAGGAACTATTGAAAC -4.00 kcal/mol Thermotoga petrophila T RKU-1, *Thermotoga naphthophila* RKU-10*, and Thermotoga* sp. RQ2

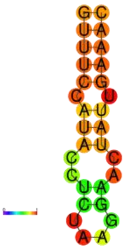

154.GTTTCCATACCTCTAAGGAAGTATTGAAAC -7.40 kcal/mol Thermotoga maritima strain Tma200, *Thermotoga maritima* strain Tma100, *Thermotoga maritima* MSB8, *Thermotoga* sp. RQ7*, and Thermotoga neapolitana* DSM 4359

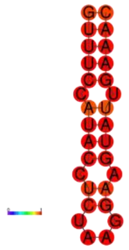

155.GTTTCCATACCTCTAAGGAATTATTGAAAC -4.00 kcal/mol Thermotoga petrophila T RKU-1, *Thermotoga maritima* strain Tma200, *Thermotoga maritima* strain Tma100, *Thermotoga maritima* MSB8, *Thermotoga naphthophila* RKU-10*, and Thermotoga* sp. RQ2

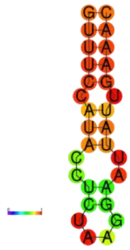

156.GTTTCCATACCTCTAAGGAATTATTGAAACC -4.80 kcal/mol Thermotoga maritima strain Tma200, *Thermotoga maritima* strain Tma100*, and Thermotoga maritima* MSB8

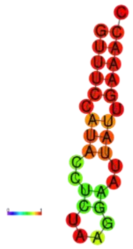

157.GTTTCCATCCCTCATAGGACCTCTCTTAAAC -1.40 kcal/mol Pseudothermotoga hypogea DSM 11164 = NBRC 106472

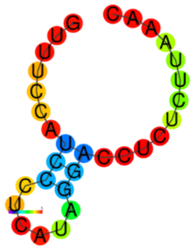

158.GTTTCCATCCCTCATAGGAGCCTTCTAAAC -1.60 kcal/mol Pseudothermotoga hypogea DSM 11164 = NBRC 106472

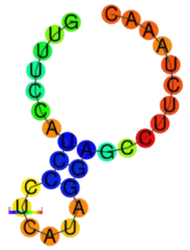

159.GTTTCCATCCCTCTAAGGTTCGATTGAAAC -5.00 kcal/mol Pseudothermotoga elfii DSM 9442 = NBRC 107921

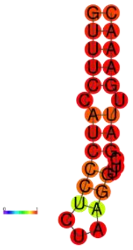

160.GTTTCCATCTCTTTCAGAGAGAACCTTCTTTCGGAC -5.80 kcal/mol Thermotoga profund a AZM34c06

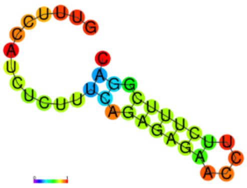

161.GTTTCCATTCCTCATAGATTCGATTGAAC 0.00 kcal/mol Pseudothermotoga hypogea DS M 11164 = NBRC 106472

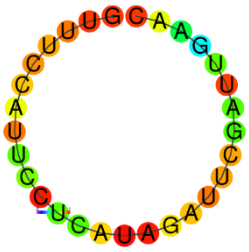

162.GTTTCCATTCCTCATAGGTAGATTCTAAAC -0.50 kcal/mol Thermosipho melanesiensis B I429 *and Thermosipho melanesiensis* strain 431

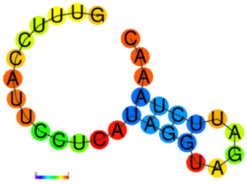

163.GTTTCCATTCCTCGTAGGTAGGCTGGGAAC -7.70 kcal/mol Thermosulfidibacter takaii ABI70S6

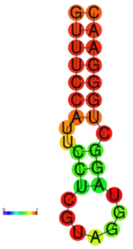

164.GTTTCCATTCCTCTTAGATTCGATTGAAAC -1.00 kcal/mol Thermotoga profunda AZM3 4c06

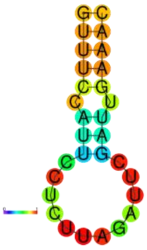

165.GTTTCGACAGTACCTATGAGGGCTTGAAAC -5.80 kcal/mol Thermomicrobium roseum DSM 5159

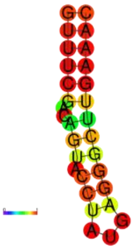

166.GTTTCTAATGTACCGTGTGGAGTTGAAAG -1.30 kcal/mol Thermocrinis albus DSM 14484

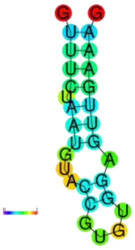

167.GTTTCTACCTTACCTTGGAGGAATTGAAAC -5.70 kcal/mol Thermosipho melanesiensis BI429 *and Thermosipho melanesiensis* strain 431

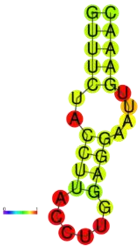

168.GTTTGAAATGCCCTATAAGGGATTGTGAC -4.30 kcal/mol Thermovibrio ammonificans HB-1

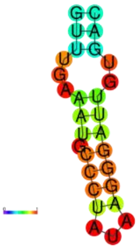

169.GTTTGAACCCTACCTATAAGGAATGGAAAC -2.00 kcal/mol Thermotoga thermarum DSM 5069

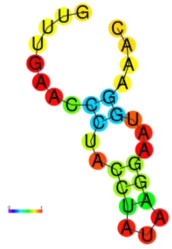

170.GTTTGATCTGAACTATGTGGGATGTGAAC -2.00 kcal/mol Pseudothermotoga hypogea DSM 11164 = NBRC 106472

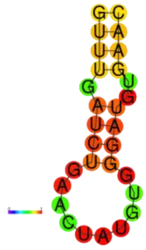

171.GTTTGGACACTACCTATGAGGAATGGAAAC -1.50 kcal/mol Thermotoga thermarum DSM 5069

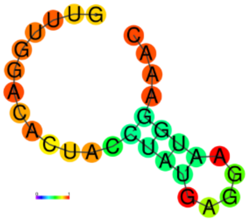

172.GTTTTAGACCTTCCTATAAGGGATGGAAAC -6.30 kcal/mol Thermovirga lienii DSM 172
91

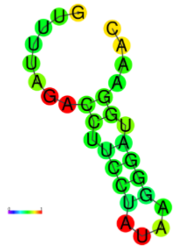

173.GTTTTATCTGAACAAGTGGGATTTAAAC -1.30 kcal/mol Thermovibrio ammonificans HB-1

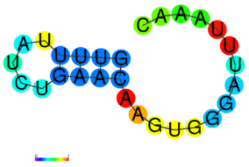

174.GTTTTATTTGAACAAGTGGGATTTAAAC -1.90 kcal/mol Thermovibrio ammonificans HB-1

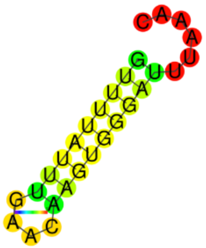

175.GTTTTCATTCCTCATAGGTAGATTCTAAAC -0.50 kcal/mol Thermosipho africanus TCF52 B

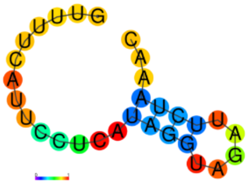

176.GTTTTGAGCCTACCTACAAGGAATTGAAAC -4.30 kcal/mol Thermodesulfobacterium com mune DSM 2178

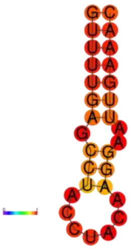

177.GTTTTGAGCCTACCTATGAGGAATTGAAAC -4.30 kcal/mol Thermodesulfovibrio yellowst onii DSM 11347

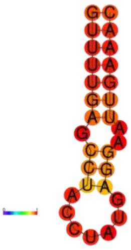

178.GTTTTGCAGCCAGTAATTGAGAGACTGAG -2.40 kcal/mol Thermoanaerobacter kivui str ain DSM 2030

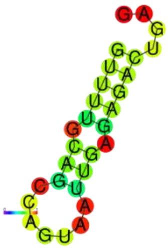

179.GTTTTTACGATACCTATGAGGAATTGAAAC -3.20 kcal/mol Thermodesulfobium narugense DSM 14796

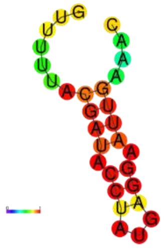

180.GTTTTTAGCCTACCTAAAAGGGATTGAAAC -4.40 kcal/mol Caldanaerobacter subterrane us subsp. tengcongensis MB4

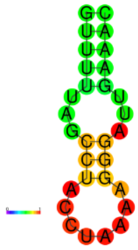

181.GTTTTTAGCCTACCTATAAGGAATTGAAAC -3.20 kcal/mol Thermoanaerobacterium ther mosaccharolyticum DSM 571

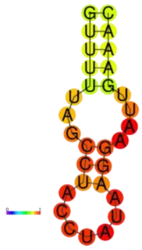

182.GTTTTTAGCCTACCTATAAGGGATTGAAAC -4.40 kcal/mol Thermosediminibacter oceani DSM 16646

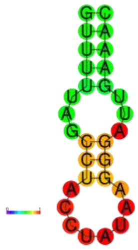

183.GTTTTTAGCCTACCTATGAGGAATTGAAAC -3.20 kcal/mol Thermoanaerobacterium sacc harolyticum JW_SL-YS485

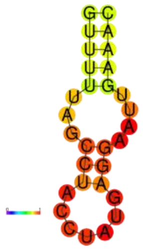

184.GTTTTTAGCTTACCTATAAGGGATTGAAAC -1.60 kcal/mol Thermoanaerobacter sp. X51 4

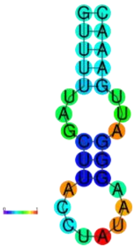

185.GTTTTTAGCTTACCTATGAGGGATTGAAAC -1.60 kcal/mol Thermosediminibacter oceani DSM 16646

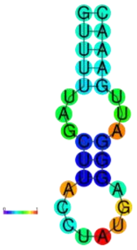

186.GTTTTTAGTCTACCTATGAGGGATTGAAAC -5.40 kcal/mol Thermoanaerobacterium sacc harolyticum JW_SL-YS485

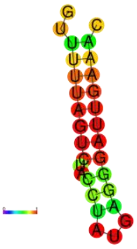

187.GTTTTTATCTTCCTAAGAGGAATATGAAC -5.40 kcal/mol Thermotoga naphthophila RKU -10*, Thermotoga sp.* RQ7*, and Thermotoga neapolitana* DSM 4359

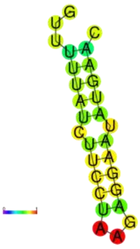

188.TAAAAGAAGCGGGTTTCCCACTTCTTTTAG -13.40 kcal/mol Thermoanaerobacter wiegeli i Rt8.B1

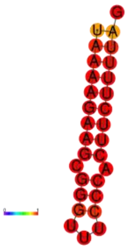

189.TATCTCTCCTACTATCTTCTTTGT 0.00 kcal/mol Thermoanaerobacter italicus Ab9

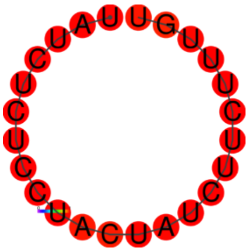

190.TCAACCCATATAGTAATTTAAAA 0.00 kcal/mol Caldanaerobacter subterraneus subsp. tengcongensis MB4

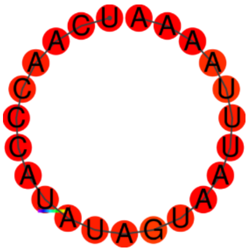

191.TCGCAATCCCTTTTAAATGAGGTCTTGTTTTCCAAC -6.10 kcal/mol Thermodesulfovibrio yellowstonii DSM 11347

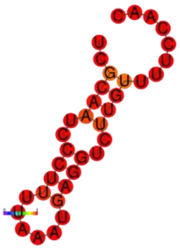

192.TCTCAGCGGGCCGCCCGCCTCAG -8.00 kcal/mol Thermobispora bispora DSM 43833

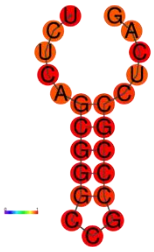

193.TCTGCGGTGGTTGCGGTGGTTTGCGGTGGTTGTG -5.40 kcal/mol Thermus scotoductus SA-01

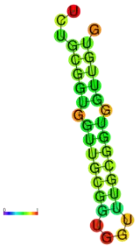

194.TGAGACTGCTTCGCTGCGCTCGCAGTGACAGG -12.30 kcal/mol Thermodesulfatator indicus DSM 15286

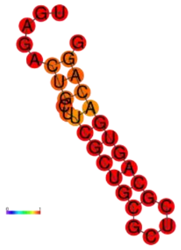

195.TGATTCCTGTACTGATTATAGTTCGA -1.40 kcal/mol Thermosediminibacter oceani DSM 16646

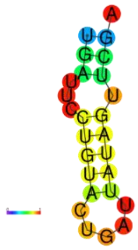

196.TTATTTTTATTTTTGTATTACATCT 0.00 kcal/mol Thermoanaerobacter brockii subsp. finnii Ako-1

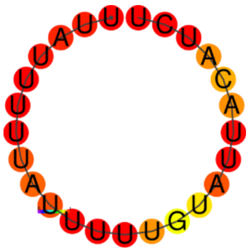

197.TTCATCACGCAAGGCACCTAGGGA 0.00 kcal/mol Thermosynechococcus elongatus BP-1

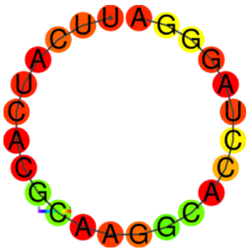

198.TTTTTAGCCTATCTATGAGGGATTGAAAC -4.00 kcal/mol Thermoanaerobacterium saccharolyticum JW_SL-YS485

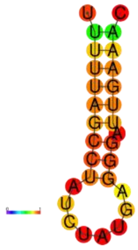

199.CGGGTGCGGGGCGCCCTGTTGGCCCGTCC -13.50 kcal/mol Thermobifida fusca YX

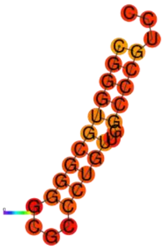

200.CGGTCCATCCCCACGTGCGTGGGGCTCAC -10.60 kcal/mol Thermobifida fusca YX

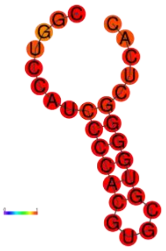

201.GACCAGGCCACCATCAACCTCGG 2.10 kcal/mol Thermobifida fusca YX

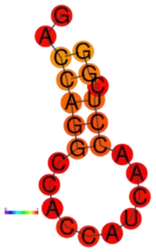

202.GAGAGCCCCACGCACGTGGGGATGGACCG -11.40 kcal/mol Thermobifida fusca YX

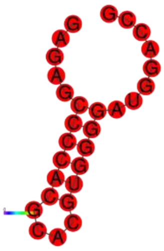

203.GGACCATCCCCGCATGCGCGGGAAGCAC -9.80 kcal/mol Thermobifida fusca YX

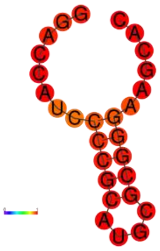

204.GGGACCATCCCCGCATGCGCGGGGAGCAC -14.30 kcal/mol Thermobifida fusca YX

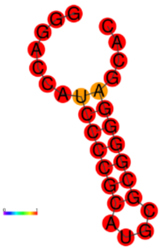

205.GGTCCATCCCCACGTGCGTGGGGAGCAT -12.80 kcal/mol Thermobifida fusca YX

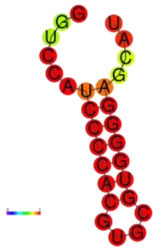

206.GGTCCATCCCCGCATGCGCGGGGAGCAC -14.30 kcal/mol Thermobifida fusca YX

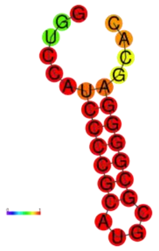

207.GTGCTCCCCACGCACGTGGGGATGGTCCG -12.80 kcal/mol Thermobifida fusca YX

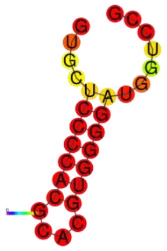

208.GTGCTCCCCGCGCATGCGGGGATGGTCC -14.30 kcal/mol Thermobifida fusca YX

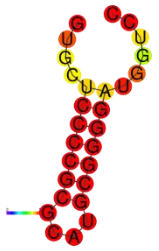

209. GTTTCCATTCCTCATAGGTATGTTCTAAAC -2.50 kcal/mol Thermosipho sp. 1063

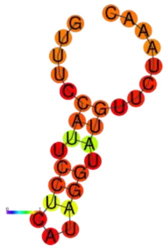

Supplement: Supplementary file 1 [file microorganisms-11-02275-s001.zip › Figure S1.pdf]
