# Supplementary material for: Comparison of Structural Features of CRISPR-Cas Systems in Thermophilic Bacteria
Source: Microorganisms. 2023 Sep 10;11(9):2275. doi: 10.3390/microorganisms11092275 (PMC10536717; doi:10.3390/microorganisms11092275)
Supplement: Supplementary file 1 [file microorganisms-11-02275-s001.zip › Table S1.pdf]

Table S1. Summary of information on the genome sequence of 61 strains of Thermophilic. bacteria, the number of CRISPR loci, the type of *cas* and optimum growth temperature

Type of Thermophilic bacteria	Sains	Optimum growth temperature	Number of CRISPR loci（Number of putative CRISPR loci）	Type of *cas*	GenBank accesion number
moderate thermophiles	*Thermomonospora curvata* DSM 43183	45 °C	14/6	IE, IE	NC_013510.1
	Thermobacillus composti KWC4	50 °C	10/3	IIID, IB, IC	NC_019897.1
	Thermobifida fusca YX	50-55 °C	13/4	IIIB，IE	NC_007333.1
	Thermobispora bispora DSM 43833	55 °C	4/16	IE	NC_014165.1
	Thermodesulfobium narugense DSM 14796	55 °C,	2/1	IB, IIID, IIIA	NC_015499.1
	Thermosynechococcus elongatus BP-1	55 °C	0/1	0 *cas*	NC_004113.1
	Thermoanaerobacterium saccharolyticum JW_SL-YS485	55 °C	5/2	IB，IB，IIID	NC_017992.1
	Thermosynechococcus sp. NK55	52-60 °C	1/0	IA	NC_023033.1
	Thermoanaerobacter brockii subsp. finnii Ako-1	55-60 °C	5/1	IB, V, IIID	NC_014964.1
	Thermoanaerobacterium thermosaccharolyticum M0795	55-60 °C	1/0	IV	NC_019956.1
	Thermotoga caldifontis AZM44c09	60 °C	2/0	ID	NZ_AP014509.1 GCF_000828655
	Thermotoga profunda AZM34c06	60 °C	3/0	IB, IIIB, IIIA, IIIC	NZ_AP014510.1 GCF_000828675
	Thermoanaerobacterium thermosaccharolyticum DSM 571	60 °C	5/0	IIIB，IB，IIID	NC_014410.1
	Thermoanaerobacterium xylanolyticum LX-11	60 °C	3/1	IB, IIID, IB	NC_015555.1
	Thermoanaerobacter sp. X513	60 °C	4/0	IIID, V, IB	NC_014538.1
	Thermoanaerobacter sp. X514	60 °C	4/0	IIID, CAS, IB	NC_010320.1
	Thermoanaerobacter kivui strain DSM 2030	61.6℃	3/4	IIIC，IIIA，IB	NZ_CP009170.1
	Thermoanaerobacter pseudethanolicus ATCC 33223	65 °C	7/1	IB，V，IIID	NC_010321.1
	Thermodesulfovibrio yellowstonii DSM 11347	65 °C	4/1	IIIA, IIIA, IIIC, IA, IB	NC_011296.1
	Thermotoga petrophila RKU-1	65 °C,	7/2	IB，IIID	NC_009486.1
	Thermus scotoductus SA-01	65 °C	3/1	IE	NC_014974.1
	Thermus thermophilus HB27	65 °C	1/1	0 *cas*	NC_005835.1
	Thermus thermophilus HB8	65 °C	2/0	0 *cas*	NC_006461.1
	Pseudothermotoga elfii DSM 9442 = NBRC 107921	66 °C	3/1	IB，IIIC	NC_022792.1
	Thermoanaerobacter wiegelii Rt8.B1	65-68 °C	2/2	IB	NC_015958.1
	Thermobaculum terrenum ATCC BAA-798	67 °C	3/2	0 *cas*	NC_013525.1
	Thermus aquaticus Y51MC23	65 -70 °C	7/3	IIIA, IIIB, no *cas1*	NZ_CP010822.1 GCF_001399775

thermophiles	*Thermosediminibacter oceani* DSM 16646	68 °C	5/2	IB, IIIA	NC_014377.1
	Thermovirga lienii DSM 17291	68 °C	2/0	IIIA, IB	NC_016148.1
	Thermus thermophilus JL-18	68 °C	0/0	0 *cas*	NC_017587.1
	Thermoanaerobacter italicus Ab9	70 °C	4/1	IIIB, IIIC, IB	NC_013921.1
	Thermodesulfobacterium commune DSM 2178	70 °C	2/1	IA	NZ_CP008796.1
	Thermodesulfatator indicus DSM 15286	70 °C，	3/2	IIIC, IB, IIIB	NC_015681.1
	Thermosipho africanus TCF52B	70℃	12/0	IIIB, IIIC, IB, IB	NC_011653.1
	Thermosipho melanesiensis BI429	70 °C	5/0	IB, IIIA	NC_009616.1
	Thermosipho melanesiensis strain 431	70 °C	5/0	IB, IIIB, IIIA	NZ_CP007389.1
	Thermosipho sp. 1063	70 °C	4/0	IIIA, ID, IIIB, IIIA	NZ_CP007223.1
	Thermosulfidibacter takaii ABI70S6	70 °C	1/0	IB	NZ_AP013035.1 GCF_001547735
	Pseudothermotoga hypogea DSM 11164 = NBRC 106472	70℃	4/0	IIIA，IIIB，IB，IB	NC_022795.1
	Thermus parvatiensis strain RL	70 °C	0/0	0 *cas*	NZ_CP014141.1
	Thermus oshimai JL-2	70 °C	4/1	IIIA，IE	NC_019386.1
	Thermus thermophilus SG0.5JP17-16	70 °C	0/2	0 *cas*	NC_017272.1
	Thermomicrobium roseum DSM 5159	70–75 °C	1/0	IB	NC_011959.1
	Thermoanaerobacter mathranii subsp. mathranii str. A3	70-75 °C	3/0	IB，IIIB，V，	NC_014209.1
	Thermovibrio ammonificans HB-1	75 °C	8/1	IIIC, IIIA, V, IB	NC_014926.1
	Caldanaerobacter subterraneus subsp. tengcongensis MB4	75℃	3/1	IIIB，IB	NC_003869.1
	Thermococcus litoralis DSM 5473	70-80 °C	7/0	IA，IB	NC_022084.1
	Thermocrinis albus DSM 14484	80 °C	4/2	IIIB，IB	NC_013894.1
	Thermotoga lettingae TMO	80℃	2/0	IB，IIIC，	NC_009828.1
	Thermotoga maritima strain Tma200	80℃	7/1	IIIB，IB，IIID	NZ_CP010967.1 GCF_000978535
	Thermotoga maritima strain Tma100	80℃	7/1	IIIB，IB，IIID	NZ_CP011108.1 GCF_000978575
	Thermotoga maritima MSB8	80℃	7/1	IIIB，IB，IIID	NZ_CP011107.1 GCF_000978555
	Thermotoga naphthophila RKU-10	80℃	8/0	IIIC，IIID	NC_013642.1
	Thermotoga sp. RQ2	80 °C	8/0	IIIC, IIID, IB, IIIB	NC_010483.1
	Thermotoga sp. RQ7	80 °C	7/1	IB	NZ_CP007633.1 GCF_000832145
	Thermotoga thermarum DSM 5069	80 °C	6/0	IB, IIIB, IIIA, IB	NC_015707.1
hyperthermophiles	*Thermodesulfobacterium geofontis* OPF15	83 °C	3/0	IIID	NC_015682.1
	Thermocrinis ruber strain DSM 23557	82-88 °C	6/3	IIIB, IB	NZ_CP007028.1
	Thermotoga neapolitana DSM 4359	85 - 90℃	7/1	IB	NC_011978.1
	Thermofilum adornatus	92℃	12/5	IIIB, IA	NC_022093.1
	Thermus sp. CCB_US3_UF1	92.4 °C	7/1	IIIB，IB，IIIA	NC_017278.1
