# Supplementary material for: Comparison of Structural Features of CRISPR-Cas Systems in Thermophilic Bacteria
Source: Microorganisms. 2023 Sep 10;11(9):2275. doi: 10.3390/microorganisms11092275 (PMC10536717; doi:10.3390/microorganisms11092275)
Supplement: Supplementary file 1 [file microorganisms-11-02275-s001.zip › Table S2.pdf]

Table S2. The CRISPR loci in 59 Thermophilic bacteria genomes.

Stain	CRISPR ID	RefSeq	CRISPR Length	DR Length	Number of repeats	Number of spacers	MFE of repeats	Optimum temperature	GC content
Thermomonospora curvata DSM 43183	NC_013510.1-1	GTTGCGATCCCTCTGAGGGGTGATGAGGACC	233	31	4	3	-7.40	45°C	61.29
	NC_013510.1-2	GTTGCGATCCCTCTGAGGGGTGATGAGGAC	291	30	5	4	-6.60	45°C	60.00
	NC_013510.1-3	GGTTGCGATCCCTCTGAGGGGTGATGAGGGC	295	31	5	4	-7.10	45°C	64.52
	NC_013510.1-4	GGGACCATCCCCGCGTGCGCGGGGAGCAG	1188	29	20	19	-14.30	45°C	79.31
	NC_013510.1-5	GTCCTCATCACCCCTCGGAGGGATCGCAAC	356	30	6	5	-4.40	45°C	63.33
	NC_013510.1-6	GTTGCGATCCCTCCAGGGGTGATGAGCGAC	356	30	6	5	-9.40	45°C	63.33
	NC_013510.1-7	GTCGCTCATCACCCCTGGAGGGATCGCAAC	1157	30	18	17	-5.00	45°C	63.33
	NC_013510.1-8	CGGACCACCCCCGCCTGCGCGGGGAGCAC	3813	29	63	62	-12.70	45°C	82.76
	NC_013510.1-9	GTGCTCCCCGCGCAGGCGGGGGTGATCCG	7718	29	127	126	-14.80	45°C	79.31
	NC_013510.1-10	GTGCTCCCCGCGCAGGCGGGGGTGATCCG	1434	29	24	23	-14.80	45°C	79.31
	NC_013510.1-11	GTGCTCCCCGCGCAGGCGGGGGTGATCCG	148	29	3	2	-14.80	45°C	79.31
	NC_013510.1-12	CCGTTCCCCGCGGGCCGGAAGGGGTG	85	26	2	1	-7.70	45°C	80.77
	NC_013510.1-13	GCGTTCTCCTGGCCCGGCAGGGCCGG	89	26	2	1	-13.80	45°C	80.77
	NC_013510.1-14	GTCCTCATCACCCCTCAGAGGGATCGCAAC	626	30	10	9	-4.50	45°C	60.00
	NC_013510.1-15	GGGATCATCCCCGCGTGCGCGGGGAGCAG	1249	29	21	20	-14.38	45°C	75.86
	NC_013510.1-16	CTGCTCCCCGCGCACGCGGGGATGGTCCC	2286	29	38	37	-14.30	45°C	79.31
	NC_013510.1-17	GCGGCGGCAGGCGCGTGAGGGGCCGCG	87	27	2	1	-10.60	45°C	88.89
	NC_013510.1-18	GTCCTCATCACCCCTCAGAGGGATCGCAAC	613	30	10	9	-4.50	45°C	60.00
	NC_013510.1-19	CGTTCACCACCAGCAGGGCCGCC	91	23	2	1	-0.80	45°C	73.91
	NC_013510.1-20	CTCCCGTCCCCGGGCCCAGCGGGC	84	24	2	1	-7.20	45°C	87.50
Thermobacillus composti KWC4	NC_019897.1-1	GCGAGGGAGGCTGCAGCGTGCAT	74	23	2	1	-3.00	50°C	69.57
	NC_019897.1-2	CTGTAACGCGGATACCGCGTTACA	75	24	2	1	-14.40	50°C	54.17

	NC_019897.1-3	GATTTAAGCGGGCGCATCCGTTTTGCGGA	86	29	2	1	-5.80	50°C	55.17
	NC_019897.1-4	GTTTCAATTCCTCATAGGTACGATCAAAAC	307	30	5	4	-0.30	50°C	36.67
	NC_019897.1-5	GTTTCAATTCCTCATAGGTACGATCAAAAC	1315	30	20	19	-0.30	50°C	36.67
	NC_019897.1-6	GTTTCAATTCCTCATAGGTACGATCAAAAC	2231	30	34	33	-0.30	50°C	36.67
	NC_019897.1-7	GTTTCAATTCCTCATAGGTACGATCAAAAC	1025	30	16	15	-0.30	50°C	36.67
	NC_019897.1-8	GTTTCAATTCCTCATAGGTACGATCAAAAC	1233	30	19	18	-0.30	50°C	36.67
	NC_019897.1-9	GTTTCAATTCCTCATAGGTACGATCAAAAC	432	30	7	6	-0.30	50°C	36.67
	NC_019897.1-10	GTTTCAATTCCTCATAGGTACGATCAAAAC	501	30	8	7	-0.30	50°C	36.67
	NC_019897.1-11	GTTTCAATTCCTCATAGGTACGATCAAAAC	297	30	5	4	-0.30	50°C	36.67
	NC_019897.1-12	GTTTCAATTCCTCATAGGTACGATCAAAAC	227	30	4	3	-0.30	50°C	36.67
	NC_019897.1-13	GTCGCCCGCTGTGCGCGGGCGTGGGTTGAAAC	2742	32	41	40	-14.60	50°C	71.88
Thermobifida fusca YX	NC_007333.1-1	GGATCATCCCCGCATGCGCGGGGAGCAC	242	28	4	3	-14.30	52.5°C	71.43
	NC_007333.1-2	CGGGTGCGGGGCGCCCTGTTGGCCCGTCC	95	29	2	1	-13.50	52.5°C	82.76
	NC_007333.1-3	GTGCTCCCCACGCACGTGGGGATGGTCCG	4058	29	67	66	-12.80	52.5°C	72.41
	NC_007333.1-4	GGTCCATCCCCACGTGCGTGGGGAGCAT	1129	28	19	18	-12.80	52.5°C	67.86
	NC_007333.1-5	GGTCCATCCCCACGTGCGTGGGGAGCAT	1179	28	21	20	-12.80	52.5°C	67.86
	NC_007333.1-6	GTGCTCCCCGCGCATGCGGGGATGGTCC	270	28	5	4	-14.30	52.5°C	75.00
	NC_007333.1-7	CGGTCCATCCCCACGTGCGTGGGGCTCAC	1680	29	28	27	-10.60	52.5°C	72.41
	NC_007333.1-8	CGGTCCATCCCCACGTGCGTGGGGCTCAC	1802	29	30	29	-10.60	52.5°C	72.41
	NC_007333.1-9	GAGAGCCCCACGCACGTGGGGATGGACCG	700	29	12	11	-11.40	52.5°C	72.41
	NC_007333.1-10	GGACCATCCCCGCATGCGCGGGAAGCAC	149	28	3	2	-9.80	52.5°C	71.43
	NC_007333.1-11	GGGACCATCCCCGCATGCGCGGGGAGCAC	272	29	5	4	-14.30	52.5°C	75.86
	NC_007333.1-12	CGGTCCATCCCCACGTGCGTGGGGCTCAC	722	29	12	11	-10.60	52.5°C	72.41
	NC_007333.1-13	CGGTCCATCCCCACGTGCGTGGGGCTCAC	273	29	5	4	-10.60	52.5°C	72.41
	NC_007333.1-14	GACCAGGCCACCATCAACCTCGG	95	23	2	1	-2.10	52.5°C	65.22

	NC_007333.1-15	GGTCCATCCCCGCATGCGCGGGGAGCAC	150	28	3	2	-14.30	52.5°C	75.00
	NC_007333.1-16	CGGTCCATCCCCACGTGCGTGGGGCTCAC	825	29	14	13	-10.60	52.5°C	72.41
	NC_007333.1-17	CGGTCCATCCCCACGTGCGTGGGGCTCAC	2592	29	43	42	-10.60	52.5°C	72.41
Thermobispora bispora DSM 43833	NC_014165.1-1	CGACATCCCGCGCTGACCGGGGGCCACGG	141	29	3	2	-7.20	55°C	79.31
	NC_014165.1-2	GTCAGTGCGGGCGGGGGAGCAGG	83	23	2	1	-1.70	55°C	78.26
	NC_014165.1-3	GTCCGGGAGGCGCGGATGAGCGCC	97	24	2	1	-9.60	55°C	79.17
	NC_014165.1-4	GTTGCGATCCCTCTAGGGGTGATGAGCGGAC	516	31	8	7	-7.60	55°C	61.29
	NC_014165.1-5	CGCGGGCCCCGCGCATGCGGGACGCGGGC	151	29	3	2	-15.50	55°C	89.66
	NC_014165.1-6	GCCACCGGGCCTCAGCGCCACCGTC	110	25	2	1	-3.80	55°C	80.00
	NC_014165.1-7	GGGATCATCCCCGCGTGCGCGGGGAGCAC	4845	29	80	79	-14.30	55°C	75.86
	NC_014165.1-8	GGGATCATCCCCGCGTGCGCGGGGAGCAC	3203	29	53	52	-14.30	55°C	75.86
	NC_014165.1-9	GGGATCATCCCCGCGTGCGCGGGGAGCAC	91	29	2	1	-14.30	55°C	75.86
	NC_014165.1-10	GCGCCCCAGGAGCACGGTGCGCGG	145	24	3	2	-9.60	55°C	83.33
	NC_014165.1-11	CGGCCCCGCATCGCCCGGGCGCCGC	106	25	2	1	-12.70	55°C	92.00
	NC_014165.1-12	GCGGAGCTTCGGGCCCCGTGGGA	95	23	2	1	-7.70	55°C	78.26
	NC_014165.1-13	GTGCTCCCCGCGCACGCGGGGATGATCCC	91	29	2	1	-14.30	55°C	75.86
	NC_014165.1-14	GTGCTCCCCGCGCACGCGGGGATGATCCC	602	29	99	98	-14.30	55°C	75.86
	NC_014165.1-15	GCGGGACCCCGGACGGGCGGGCGG	104	24	2	1	-6.50	55°C	91.67
	NC_014165.1-16	CGACCGGCGGCCCGCTCACGGCC	84	23	2	1	-5.40	55°C	86.96
	NC_014165.1-17	CGGCGGCGCGCGGACACGACGGGTGA	87	26	2	1	-6.30	55°C	80.77
	NC_014165.1-18	GAGGTCGCCGGTGTTCATCGCCATC	84	25	2	1	-4.60	55°C	64.00
	NC_014165.1-19	ATGGCCCGGAAACGCGCGGGGCG	137	23	3	2	-9.20	55°C	78.26
	NC_014165.1-20	TCTCAGCGGGCCGCCCGCCTCAG	96	23	2	1	-8.00	55°C	78.26
Thermodesulfobium narugense DSM 14796	NC_015499.1-1	GTTTTTACGATACCTATGAGGAATTGAAAC	5642	30	85	84	-3.20	55°C	33.33
	NC_015499.1-2	AGTTTTTACGATACCTATGAGGAATTGAAACA	96	32	2	1	-3.90	55°C	31.25

	NC_015499.1-3	GTCGCAATCCCTTATTCTTCAGGGAATTTTTCTAAC	7184	36	95	94	-6.00	55°C	38.89
Thermosynechococcus elongatus BP-1	NC_004113.1-1	TTCATCACGCAAGGCACCTAGGGA	88	24	2	1	0.00	55°C	54.17
Thermoanaerobacterium saccharolyticum JW_SL-YS485	NC_017992.1-1	GTTTCAATTCCTTATAGGTAGGCTAAAAAC	3687	30	56	55	-0.80	55°C	33.33
	NC_017992.1-2	GTTTATAACCCACAATGGTTCTACCTAAAC	359	30	6	5	-1.80	55°C	36.67
	NC_017992.1-3	GTTTTTAGTCTACCTATGAGGGATTGAAAC	1234	30	19	18	-5.40	55°C	36.67
	NC_017992.1-4	GTTTTTAGCCTACCTATAAGGAATTGAAAC	900	30	14	13	-3.20	55°C	33.33
	NC_017992.1-5	TTTTTAGCCTATCTATGAGGGATTGAAAC	96	29	2	1	-4.00	55°C	34.48
	NC_017992.1-6	GTTTTTAGCCTACCTATGAGGAATTGAAAC	1102	30	17	16	-3.20	55°C	36.67
	NC_017992.1-7	CCCTTTTCACCATAAAATCAATACTTTTCAAC	99	32	2	1	0.00	55°C	31.25
Thermosynechococcus sp. NK55	NC_023033.1-1	GTGCTCAACGCCTTACGGCATCAGAGGTAGAGGCAC	1750	36	25	24	-9.00	56°C	58.33
Thermoanaerobacter brockii subsp. finnii Ako-1	NC_014964.1-1	TTATTTTTATTTTTGTATTACATCT	187	25	3	2	0.00	57.5°C	12.00
	NC_014964.1-2	GTTTTTAGCCTACCTATAAGGAATTGAAAC	3238	30	49	48	-3.20	57.5°C	33.33
	NC_014964.1-3	GTTTTTAGCCTACCTATAAGGAATTGAAAC	1501	30	23	22	-3.20	57.5°C	33.33
	NC_014964.1-4	GTTTTTAGCCTACCTATAAGGAATTGAAAC	2239	30	34	33	-3.20	57.5°C	33.33
	NC_014964.1-5	GTTTTTAGCCTACCTATAAGGAATTGAAAC	1699	30	26	25	-3.20	57.5°C	33.33
	NC_014964.1-6	GTTTTTAGCCTACCTATAAGGAATTGAAAC	1300	30	20	19	-3.20	57.5°C	33.33
Thermoanaerobacterium M0795	NC_019956.1-1	CTTCTAAACCTACATAGGATATTTCAAAC	619	29	10	9	-1.30	57.5°C	31.03
Thermotoga caldifontis AZM44c09	NZ_AP014509.1-1	CTTTCAATTCCGTTCTACGGAATTTGGTCTTGAGGC	711	36	10	9	-7.70	60°C	44.44
	NZ_AP014509.1-2	GTTTCAATCGAACCTTAGAGGGATGGAAAC	1974	30	30	29	-5.00	60°C	43.33
Thermotoga profunda AZM34c06	NZ_AP014510.1-1	GTTTCCATTCCTCTTAGATTCGATTGAAAC	5886	30	89	88	-1.00	60°C	36.67
	NZ_AP014510.1-2	GTTTCCATCTCTTTCAGAGAGAACCTTCTTTCGGAC	2916	36	39	38	-5.80	60°C	44.44
	NZ_AP014510.1-3	GTTTCCATCTCTTTCAGAGAGAACCTTCTTTCGGAC	2704	36	36	35	-5.80	60°C	44.44

Thermoanaerobacterium thermosaccharolyticum DSM 571	NC_014410.1-1	GTTTTTAGCCTACCTATAAGGGATTGAAAC	2720	30	41	40	-4.40	60°C	36.67
	NC_014410.1-2	GTTTTTAGCCTACCTATGAGGAATTGAAAC	294	30	5	4	-3.20	60°C	36.67
	NC_014410.1-3	GTTTTTAGCCTACCTATGAGGAATTGAAAC	1033	30	16	15	-3.20	60°C	36.67
	NC_014410.1-4	GTTTCAATTCCTGATAGGTAGGCTAAAAAC	3899	30	59	58	-1.50	60°C	36.67
	NC_014410.1-5	GTTTCAATTCCACTATGGTTAGATTAAATC	2682	30	41	40	-0.20	60°C	30.00
Thermoanaerobacterium xylanolyticum LX-11	NC_015555.1-1	GTTGAAAAGTATTGATATTATGTCGAGAAGG	97	31	2	1	-1.90	60°C	32.26
	NC_015555.1-2	GTTTCAATTCCTTATAGGTAGGCTAAAAAC	5628	30	85	84	-0.80	60°C	33.33
	NC_015555.1-3	GTTTCAATTCCACTATGGTTAGATTAAATC	859	30	13	12	-0.20	60°C	30.00
	NC_015555.1-4	GTTTCAATTCCACTATGGTTAGATTAAATC	9232	30	140	139	-0.20	60°C	30.00
Thermoanaerobacter sp. X513	NC_014538.1-1	GTTTCAATCCCTTATAGGTAAGCTAAAAAC	362	30	6	5	-0.50	60°C	33.33
	NC_014538.1-2	GTTTCAATTCCTTATAGGTAGGCTAAAAAC	700	30	11	10	-0.80	60°C	33.33
	NC_014538.1-3	GTTTCAATTCCTTATAGGTAGGCTAAAAAC	4138	30	63	62	-0.80	60°C	33.33
	NC_014538.1-4	GTTTCAATTCCTTATAGGTAGGCTAAAAAC	13650	30	206	205	-0.80	60°C	33.33
Thermoanaerobacter sp. X514	NC_010320.1-1	GTTTTTAGCTTACCTATAAGGGATTGAAAC	362	30	6	5	-1.60	60°C	33.33
	NC_010320.1-2	GTTTCAATTCCTTATAGGTAGGCTAAAAAC	700	30	11	10	-0.80	60°C	33.33
	NC_010320.1-3	GTTTCAATTCCTTATAGGTAGGCTAAAAAC	4138	30	63	62	-0.80	60°C	33.33
	NC_010320.1-4	GTTTCAATTCCTTATAGGTAGGCTAAAAAC	14581	30	220	219	-0.80	60°C	33.33
Thermoanaerobacter kivui strain DSM 2030	NZ_CP009170.1-1	CAGTTCTAGCAGTTAGAACGAGTTGTAAACCT	96	32	2	1	-5.10	61.6°C	40.62
	NZ_CP009170.1-2	GTTTTGCAGCCAGTAATTGAGAGACTGAG	94	29	2	1	-2.40	61.6°C	44.83
	NZ_CP009170.1-3	CGCCGTCGTTTTTATAAGGGGAATAGAAACCC	98	32	2	1	-3.90	61.6°C	46.88
	NZ_CP009170.1-4	GTTTCAATTCCTTATAGGTAGGCTAAAAAC	4555	30	69	68	-0.80	61.6°C	33.33
	NZ_CP009170.1-5	GTTTCAATCCCTCTTAGGTAGGCTAAAAAC	5697	30	86	85	-1.30	61.6°C	40.00
	NZ_CP009170.1-6	CTTTCAATTCCAGTATGGTTGGATTAAATC	2484	30	37	36	-3.10	61.6°C	33.33
	NZ_CP009170.1-7	GTTCTACTGTAACTTAGAAGTTTTTTGTG	94	29	2	1	-1.70	61.6°C	31.03
Thermoanaerobacter	NC_010321.1-1	TTATTTTTATTTTTGTATTACATCT	187	25	3	2	0.00	65°C	12.00

pseudethanolicus ATCC 33223	NC_010321.1-2	GTTTTTAGCCTACCTATAAGGAATTGAAAC	3238	30	49	48	-3.20	65°C	33.33
	NC_010321.1-3	GTTTTTAGCCTACCTATAAGGAATTGAAAC	1501	30	23	22	-3.20	65°C	33.33
	NC_010321.1-4	GTTTTTAGCCTACCTATAAGGAATTGAAAC	3181	30	48	47	-3.20	65°C	33.33
	NC_010321.1-5	GTTTTTAGCCTACCTATAAGGAATTGAAAC	827	30	13	12	-3.20	65°C	33.33
	NC_010321.1-6	GTTTTTAGCCTACCTATAAGGAATTGAAAC	896	30	14	13	-3.20	65°C	33.33
	NC_010321.1-7	GTTTTTAGCCTACCTATAAGGAATTGAAAC	1699	30	26	25	-3.20	65°C	33.33
	NC_010321.1-8	GTTTTTAGCCTACCTATAAGGAATTGAAAC	1300	30	20	19	-3.20	65°C	33.33
Thermodesulfovibrio yellowstonii DSM 11347	NC_011296.1-1	TCGCAATCCCTTTTAAATGAGGTCTTGTTTTCCAAC	262	36	4	3	-6.10	65°C	38.89
	NC_011296.1-2	GTAGGAAAACAAGACCTCATTTAAAAGGGATTGCGAC	1888	36	27	26	-5.30	65°C	41.67
	NC_011296.1-3	GTTTTGAGCCTACCTATGAGGAATTGAAAC	2093	30	32	31	-4.30	65°C	40.00
	NC_011296.1-4	GTTGGAAAACAAGACCTCATTTAAAAGGGATT	140	32	3	2	-2.60	65°C	34.38
	NC_011296.1-5	CTTTATATCCCACATGGTTCAGATGAAAC	7918	29	121	120	-0.60	65°C	37.93
Thermotoga petrophila T RKU-1	NC_009486.1-1	GTTTCAATAGTTCCTTAGAGGTATGGAAAC	299	30	5	4	-6.70	65°C	36.67
	NC_009486.1-2	GTTTCCATACCTCTAAGGAACTATTGAAAC	829	30	13	12	-4.00	65°C	36.67
	NC_009486.1-3	GTTTCCATACCTCTAAGGAACTATTGAAAC	494	30	8	7	-4.00	65°C	36.67
	NC_009486.1-4	GTTTCCATACCTCTAAGGAATTATTGAAAC	1182	30	19	18	-4.00	65°C	33.33
	NC_009486.1-5	GTTCATATTCCTCTTAGGAAGATAAAAAC	2585	29	40	39	-3.10	65°C	31.03
	NC_009486.1-6	GTTTCAATAATTCCTTAGAGGTATGGAAAC	825	30	13	12	-4.10	65°C	33.33
	NC_009486.1-7	GGTTTTTTCAATTCGAATAATCGAAAGAAC	95	30	2	1	-4.30	65°C	30.00
	NC_009486.1-8	GTTTCCATACCTCTAAGGAATTATTGAAAC	163	30	3	2	-4.00	65°C	33.33
	NC_009486.1-9	GTTTCAATAATTCCTTAGAGGTATGGAAAC	434	30	7	6	-4.10	65°C	33.33
Thermus scotoductus SA-01	NC_014974.1-1	AGTAGTCCCCACGCACGTGGGGATGGCCCG	577	30	10	9	-15.50	65°C	70.00
	NC_014974.1-2	CGGGCCATCCCCACGCGTGTGGGGACTAC	2230	29	37	36	-12.60	65°C	72.41
	NC_014974.1-3	GTAGTCCCCACGCATGTGGGGATGGCCCG	3705	29	43	42	-15.48	65°C	68.97
	NC_014974.1-4	TCTGCGGTGGTTGCGGTGGTTTGCGGTGGTTGTG	107	34	2	1	-5.40	65°C	61.76

Thermus thermophilus HB27	NC_005835.1-1	GTTGCAAGGGATTGAGCCCCGTAAGGGGATTGCGAC	163	36	3	2	-11.70	65°C	58.33
	NC_005835.1-2	GTCGCAATCCCCTTACGGGGCTCAATCCCTTGCAAC	782	36	7	6	-7.40	65°C	58.33
Thermus thermophilus HB8	NC_006461.1-1	GTTGCAAGGGATTGAGCCCCGTAAGGGGATTGCGAC	1100	36	15	14	-11.70	65°C	58.33
	NC_006461.1-2	GTTGCAAGGGATTGAGCCCCGTAAGGGGATTGCGAC	338	36	5	4	-11.70	65°C	58.33
Pseudothermotoga elfii DSM 9442 = NBRC 107921	NC_022792.1-1	GTTTCCATCCCTCTAAGGTTCGATTGAAAC	8794	30	133	132	-5.00	66 °C	43.33
	NC_022792.1-2	GTTTCAATCGAACCTTAGAGGGATGGAAAC	5033	30	76	75	-5.00	66 °C	43.33
	NC_022792.1-3	GTTTCAATCGAACCTTAGAGGGATGGAAAC	1220	30	19	18	-5.00	66 °C	43.33
	NC_022792.1-4	AGTCCGAAAGAAGGTCCTCTCTGAAAGAGATGG	105	33	2	1	-5.10	66 °C	48.48
Thermoanaerobacter wiegelii Rt8.B1	NC_015958.1-1	TAAAAGAAGCGGGTTTCCCACTTCTTTTAG	109	30	2	1	-13.40	66.5°C	40.00
	NC_015958.1-2	ACTATTTTCAGGATAGGTAGGCTAAAAAC	96	29	2	1	-2.40	66.5°C	34.48
	NC_015958.1-3	CTTTCAATTCCTTATAGGTAGGCTAAAAAC	2744	30	42	41	-0.80	66.5°C	33.33
	NC_015958.1-4	GTTTCAATTCCTTATAGGTAGGCTAAAAAC	5734	30	87	86	-0.80	66.5°C	33.33
Thermobaculum terrenum ATCC BAA-798	NC_013525.1-1	ATTGTCCCCACACGCGTGGGGGTGTACACCG	1128	29	19	18	-11.70	67°C	64.52
	NC_013525.1-2	CTATAGTTGTTGGTTGTTGGTTTT	81	24	2	1	0.00	67°C	33.33
	NC_013525.1-3	CGGTTCACCCCCACGCGTGTGGGGACAAT	761	29	13	12	-11.60	67°C	65.52
	NC_013525.1-4	CGTGCAGGCCTCGTATCTGCTCTGAGGTGCAAG	102	33	2	1	-8.10	67°C	60.61
	NC_013525.1-5	ATTGTCCCCACACGCGTGGGGGTGTACACCG	639	31	11	10	-11.70	67°C	64.52
Thermus aquaticus Y51MC23	NZ_CP010822.1-1	GTCGCAATCCCCTTACGGGGCTAAGTGGTTTGCAAC	450	36	7	6	-8.40	67.5°C	55.56
	NZ_CP010822.1-2	ATCATCTGACTACCTGACTACCG	98	23	2	1	0.00	67.5°C	47.83
	NZ_CP010822.1-3	GGGCCATCCCCACGTGTGTGGGGACT	151	26	3	2	-12.60	67.5°C	69.23
	NZ_CP010822.1-4	GGGCCATCCCCACGTGTGTGGGGACT	210	26	4	3	-12.60	67.5°C	69.23
	NZ_CP010822.1-5	CTTTTGACCGTACCTATGAGGGTTTGAAAC	1504	30	23	22	-3.80	67.5°C	43.33
	NZ_CP010822.1-6	GTCGCAATCCCCTTACGGGGCTAAGTGGTTTGCAAC	114	36	2	1	-8.40	67.5°C	55.56
	NZ_CP010822.1-7	GTCGCAATCCCCTTACGGGGCTAAGTGGTTTGCAAC	267	36	4	3	-8.40	67.5°C	55.56

	NZ_CP010822.1-8	GTCGCAATCCCCTTACGGGGCTAAGTGG	948	28	13	12	-8.90	67.5°C	60.71
	NZ_CP010822.1-9	GTCGCAATCCCCTTACGGGGCTAAGTGGTTTGCAAC	556	36	8	7	-8.40	67.5°C	55.56
	NZ_CP010822.1-10	GTCGCAATCCCCTTACGGGGCTAAGTGGTTTGCAAC	407	36	6	5	-8.40	67.5°C	55.56
Thermosediminibacter oceani DSM 16646	NC_014377.1-1	GTTACTAGCTTACCTATGAGGGGTTGAAAC	4700	30	70	69	-2.90	68°C	43.33
	NC_014377.1-2	GTTACTAGCTTACCTATGAGGGGTTGAAACAT	99	32	2	1	-3.50	68°C	40.62
	NC_014377.1-3	GTTTTTAGCTTACCTATGAGGGATTGAAAC	2779	30	42	41	-1.60	68°C	36.67
	NC_014377.1-4	GTTTTTAGCCTACCTATAAGGGATTGAAAC	1701	30	26	25	-4.40	68°C	36.67
	NC_014377.1-5	GTTTTTAGCCTACCTATAAGGGATTGAAAC	433	30	7	6	-4.40	68°C	36.67
	NC_014377.1-6	TGATTCCTGTACTGATTATAGTTCGA	107	26	2	1	-1.40	68°C	34.62
	NC_014377.1-7	GTTCTTAGTCTACCTATAAGGGATTGAAAC	1030	30	16	15	-3.90	68°C	36.67
Thermovirga lienii DSM 17291	NC_016148.1-1	GATTCAATCGAACCGATACGGAATGGAAAC	1506	30	24	23	-2.60	68°C	43.33
	NC_016148.1-2	GTTTTAGACCTTCCTATAAGGGATGGAAAC	3008	30	46	45	-6.30	68°C	40.00
Thermoanaerobacter italicus Ab9	NC_013921.1-1	GTTTTTAGCCTACCTATAAGGAATTGAAAC	3697	30	56	55	-3.20	70°C	33.33
	NC_013921.1-2	CACTATTTTCAGGATAGGTAGGCTAAAAAC	1900	30	29	28	-2.40	70°C	36.67
	NC_013921.1-3	TATCTCTCCTACTATCTTCTTTGT	102	24	2	1	0.00	70°C	33.33
	NC_013921.1-4	GTTTCAATTCCTCATAGGTAGGCTAAAAAC	301	30	5	4	-0.20	70°C	36.67
	NC_013921.1-5	GTTTCAATTCCTCATAGGTAGGCTAAAAAC	1289	30	20	19	-0.20	70°C	36.67
Thermodesulfobacterium commune DSM 2178	NZ_CP008796.1-1	CTTTATATCTCACATGGTTCAGATGAAAC	160	29	3	2	-0.40	70°C	34.48
	NZ_CP008796.1-2	GTAGAAACCTGCCCTACTTCAAAAGGGATTGCGAC	934	35	13	12	-5.00	70°C	48.57
	NZ_CP008796.1-3	GTTTTGAGCCTACCTACAAGGAATTGAAAC	1433	30	22	21	-4.30	70°C	40.00
Thermodesulfatator indicus DSM 15286	NC_015681.1-1	GTTCACAGCCTAACTAAAAGGAATGGAAAC	3909	30	59	58	-3.50	70°C	40.00
	NC_015681.1-2	TGAGACTGCTTCGCTGCGCTCGCAGTGACAGG	100	32	2	1	-12.30	70°C	62.50
	NC_015681.1-3	CTCGCAGTGACAAATGTGATCAGGG	151	25	3	2	-4.40	70°C	52.00
	NC_015681.1-4	GTGAGAAAACCTTGCCTGATTAAGAAGGCATTACGAC	1736	37	23	22	-7.40	70°C	43.24

	NC_015681.1-5	GTGAGAAAACCTTGCCTGATTAAGAAGGCATTACGAC	3673	37	48	47	-7.40	70°C	43.24
Thermosipho africanus TCF52B	NC_011653.1-1	GTTTAGAATCTACCTATGAGGAATGAAAAC	729	30	11	10	-0.60	70°C	33.33
	NC_011653.1-2	GTTTAGAATCTACCTATGAGGAATGAAAAC	1288	30	19	18	-0.60	70°C	33.33
	NC_011653.1-3	GTTTTCATTCCTCATAGGTAGATTCTAAAC	2406	30	35	34	-0.50	70°C	33.33
	NC_011653.1-4	GTTTAGAATCTACCTATGAGGAATGAAAAC	3208	30	46	45	-0.60	70°C	33.33
	NC_011653.1-5	GTTTTCATTCCTCATAGGTAGATTCTAAAC	2656	30	38	37	-0.50	70°C	33.33
	NC_011653.1-6	GTTTTCATTCCTCATAGGTAGATTCTAAAC	2292	30	33	32	-0.50	70°C	33.33
	NC_011653.1-7	GTTTTCATTCCTCATAGGTAGATTCTAAAC	575	30	9	8	-0.50	70°C	33.33
	NC_011653.1-8	ATTTCAATTCCTGCAAGGTAAGGTACAAAC	1431	30	22	21	-2.10	70°C	36.67
	NC_011653.1-9	GTTTCAATTCCTACAAGGTAAGGTACAAAC	1430	30	22	21	-1.20	70°C	36.67
	NC_011653.1-10	GTTTCAATCCCTAATAGGTATGCTAAAAAC	2018	30	31	30	-0.50	70°C	33.33
	NC_011653.1-11	GTTTTCATTCCTCATAGGTAGATTCTAAAC	783	30	12	11	-0.50	70°C	33.33
	NC_011653.1-12	ATTTCAATTCCTACAAGGTAAGGTACAAAC	3629	30	55	54	-1.10	70°C	33.33
Thermosipho melanesiensis BI429	NC_009616.1-1	GTTTCTACCTTACCTTGGAGGAATTGAAAC	634	30	10	9	-5.70	70°C	40.00
	NC_009616.1-2	ATTTCAATTCCTCCAAGGTAAGGTAAAAAC	499	30	8	7	-0.80	70°C	33.33
	NC_009616.1-3	ATTTCTATTCCTCATAGGTAGATTCTAAAC	3607	30	52	51	-2.30	70°C	30.00
	NC_009616.1-4	GTTTAGAATCTACCTATGAGGAATGGAAAC	1086	30	16	15	-2.00	70°C	36.67
	NC_009616.1-5	GTTTCCATTCCTCATAGGTAGATTCTAAAC	872	30	13	12	-0.50	70°C	36.67
Thermosipho melanesiensis strain 431	NZ_CP007389.1-1	GTTTCTACCTTACCTTGGAGGAATTGAAAC	634	30	10	9	-5.70	70°C	40.00
	NZ_CP007389.1-2	ATTTCAATTCCTCCAAGGTAAGGTAAAAAC	499	30	8	7	-0.80	70°C	33.33
	NZ_CP007389.1-3	ATTTCTATTCCTCATAGGTAGATTCTAAAC	3881	30	56	55	-2.30	70°C	30.00
	NZ_CP007389.1-4	GTTTAGAATCTACCTATGAGGAATGGAAAC	1086	30	16	15	-2.00	70°C	36.67
	NZ_CP007389.1-5	GTTTCCATTCCTCATAGGTAGATTCTAAAC	872	30	13	12	-0.50	70°C	36.67
Thermosipho sp. 1063	NZ_CP007223.1-1	GTTTCCATTCCTCATAGGTATGTTCTAAAC	568	30	9	8	-2.50	70°C	36.67

	NZ_CP007223.1-2	GTTAAAAAACCTAATTCCATAAATGGAATTCAAAC	4313	35	61	60	-5.20	70°C	25.71
	NZ_CP007223.1-3	GTTTAGAACATACCTATGAGGAATGGAAAC	784	30	12	11	-3.10	70°C	36.67
	NZ_CP007223.1-4	GTTTCCATTCCTCATAGGTATGTTCTAAAC	1840	30	28	27	-2.50	70°C	36.67
Thermosulfidibacter takaii ABI70S6	NZ_AP013035.1-1	GTTTCCATTCCTCGTAGGTAGGCTGGGAAC	4807	30	73	72	-7.70	70°C	53.33
Pseudothermotoga hypogea DSM 11164 = NBRC 106472	NC_022795.1-1	GTTTCCATCCCTCATAGGAGCCTTCTAAAC	1014	30	15	14	-1.60	70°C	46.67
	NC_022795.1-2	GTTTCCATCCCTCATAGGACCTCTCTTAAAC	814	30	12	11	-1.40	70°C	45.16
	NC_022795.1-3	GTTTCCATTCCTCATAGATTCGATTGAAC	5077	29	78	77	0.00	70°C	37.93
	NC_022795.1-4	GTTTGATCTGAACTATGTGGGATGTGAAC	1945	29	30	29	-2.00	70°C	41.38
Thermus oshimai JL-2	NC_019386.1-1	GTCGCAATCCCCTGACGGGGAAGCATCTCGTGCAAC	1129	36	15	14	-10.90	70°C	61.11
	NC_019386.1-2	GTCGCAATCCCCTGACGGGGAAGCATCTCGTGCAAC	943	36	7	6	-10.90	70°C	61.11
	NC_019386.1-3	CGGTCCATCCCCACGGGCGTGGGGACTAC	5387	29	89	88	-12.60	70°C	72.41
	NC_019386.1-4	GTAGTCCCCACACGCGTGGGGATGGACCG	1856	29	31	30	-14.30	70°C	68.97
	NC_019386.1-5	GTTGCACGAGATGCTTCCCCGTCAGGGGATTGCGACC	186	37	3	2	-13.30	70°C	62.16
Thermus thermophilus SG0.5JP17-16	NC_017272.1-1	CCATCTCCGCCTTGAGCTCCTGGCGCA	159	27	3	2	-5.40	70°C	66.67
	NC_017272.1-2	CCAGGGCCGCCTGGGCGAGGAGGA	106	24	2	1	-8.70	72.5°C	79.17
Thermomicrobium roseum DSM 5159	NC_011959.1-1	GTTTCGACAGTACCTATGAGGGCTTGAAAC	5067	30	77	76	-5.80	72.5°C	46.67
Thermoanaerobacter mathranii subsp. mathranii str. A3	NC_014209.1-1	ATTTACATCCCTCATAGTTCAGATAAAAC	3646	29	56	55	0.00	72.5°C	31.03
	NC_014209.1-2	GTTTTTAGCCTACCTATAAGGAATTGAAAC	1958	30	30	29	-3.20	72.5°C	33.33
	NC_014209.1-3	GTTTCAATTCCTTATAGGTAGGCTAAAAAC	1570	30	24	23	-0.80	75°C	33.33
Thermovibrio ammonificans HB-1	NC_014926.1-1	AGGTTTGTTGCTTCATGTGTGTAAGAAAA	98	29	2	1	-2.90	75°C	34.48
	NC_014926.1-2	GTTTGAAATGCCCTATAAGGGATTGTGAC	425	29	7	6	-4.30	75°C	41.38
	NC_014926.1-3	GTTGAAATGCCCGAAAAAATCGGCGGGATTGCGAC	805	35	11	10	-8.10	75°C	51.43
	NC_014926.1-4	GTTTTATCTGAACAAGTGGGATTTAAAC	223	28	4	3	-1.30	75°C	32.14

	NC_014926.1-5	GTTTAAATCCCACTTGTTCAAATAAAAC	1581	28	25	24	0.00	75°C	28.57
	NC_014926.1-6	GTTGAAATGCCCGAAAAAGTAGACGGGATTGCGAC	1109	35	15	14	-7.90	75°C	48.57
	NC_014926.1-7	GTCGCAATCCCGTCTACTTTTTCGGGCATTTCAAC	341	35	5	4	-4.80	75°C	48.57
	NC_014926.1-8	GTTTAAATCCCACTTGTTCAGATAAAAC	616	28	10	9	0.00	75°C	32.14
	NC_014926.1-9	GTTTTATTTGAACAAGTGGGATTTAAAC	617	28	10	9	-1.90	75°C	28.57
Caldanaerobacter subterraneus subsp. tengcongensis MB4	NC_003869.1-1	TCAACCCATATAGTAATTTAAAA	90	23	2	1	0.00	75°	21.74
	NC_003869.1-2	GTTTTTAGCCTACCTAAAAGGGATTGAAAC	4372	30	66	65	-4.40	75°C	36.67
	NC_003869.1-3	GTTTCAATCCCTCTTAGGTAGGCTAAAAAC	1729	30	26	25	-1.30	75°C	40.00
	NC_003869.1-4	GTTTCAATCCCTCTTAGGTAGGCTAAAAAC	14512	30	217	216	-1.30	75°C	40.00
Thermococcus litoralis DSM 5473	NC_022084.1-1	CTTTCAATTCTTCTAAAGTCTTATTGGAAC	2751	30	41	40	-2.00	75°C	30.00
	NC_022084.1-2	GTTCCAATAAGACTTTAAAAGAATTGAAAG	3403	30	51	50	0.00	75°C	26.67
	NC_022084.1-3	GTTCCAATAAGACTTTAAAAGAATTGAAAG	2365	30	35	34	0.00	75°C	26.67
	NC_022084.1-4	CTTTCAATTCTTTTAAAGTCTTATTGGAAC	643	30	10	9	-2.00	75°C	26.67
	NC_022084.1-5	CTTTCAATTCTTTTAAAGTCTTATTGGAAC	3181	30	47	46	-2.00	75°C	26.67
	NC_022084.1-6	GTTCCAATAAGACTTTAAAAGAATTGAAAG	4664	30	69	68	0.00	75°C	26.67
	NC_022084.1-7	CTTTCAATTCTTTTAAAGTCTTATTGGAAC	363	30	6	5	-2.00	75°C	26.67
Thermocrinis albus DSM 14484	NC_013894.1-1	CAACTCCACACGGTACATTAGAAAC	90	25	2	1	0.00	80°C	44.00
	NC_013894.1-2	CTTTCAACTCCACACGGTACATTAGAAAC	2694	29	41	40	0.00	80°C	41.38
	NC_013894.1-3	GTTTCTAATGTACCGTGTGGAGTTGAAAG	3794	29	57	56	-1.30	80°C	41.38
	NC_013894.1-4	GTTTCTAATGTACCGTGTGGAGTTGAAAG	2380	29	36	35	-1.30	80°C	41.38
	NC_013894.1-5	ATCAATCAATTACTTATACATCTAA	97	25	2	1	0.00	80°C	20.00
	NC_013894.1-6	CTTTCAACTCCACACGGTACATTAGAAAC	4081	29	61	60	0.00	80°C	41.38
Thermotoga lettingae TMO	NC_009828.1-1	GTTTCCATCCCTCTAAGGTTCGATTGAAAC	2888	30	44	43	-5.00	80°C	43.33
	NC_009828.1-2	GTTTCAATCGAACCTTAGAGGGATGGAAAC	3542	30	54	53	-5.00	80°C	43.33
Thermotoga maritima	NZ_CP010967.1-1	GTTTCAATAATTCCTTAGAGGTATGGAAAC	2694	30	41	40	-4.10	80°C	33.33

strain Tma200	NZ_CP010967.1-2	GTTTCAATAATTCCTTAGAGGTATGGAAAC	562	30	9	8	-4.10	80°C	33.33
	NZ_CP010967.1-3	GTTTCAATAATTCCTTAGAGGTATGGAAAC	566	30	9	8	-4.10	80°C	33.33
	NZ_CP010967.1-4	GTTTCCATACCTCTAAGGAATTATTGAAAC	1629	30	25	24	-4.00	80°C	33.33
	NZ_CP010967.1-5	GTTTCCATACCTCTAAGGAATTATTGAAACC	162	31	3	2	-4.80	80°C	35.48
	NZ_CP010967.1-6	GTTTCAATACTTCCTTAGAGGTATGGAAAC	563	30	9	8	-10.30	80°C	36.67
	NZ_CP010967.1-7	GTTTCCATACCTCTAAGGAAGTATTGAAAC	227	30	4	3	-7.40	80°C	36.67
	NZ_CP010967.1-8	GTTTCCATACCTCTAAGGAATTATTGAAAC	892	30	14	13	-4.00	80°C	33.33
Thermotoga maritima strain Tma100	NZ_CP011108.1-1	GTTTCAATAATTCCTTAGAGGTATGGAAAC	2694	30	41	40	-4.10	80°C	33.33
	NZ_CP011108.1-2	GTTTCAATAATTCCTTAGAGGTATGGAAAC	562	30	9	8	-4.10	80°C	33.33
	NZ_CP011108.1-3	GTTTCAATAATTCCTTAGAGGTATGGAAAC	566	30	9	8	-4.10	80°C	33.33
	NZ_CP011108.1-4	GTTTCCATACCTCTAAGGAATTATTGAAAC	1629	30	25	24	-4.00	80°C	33.33
	NZ_CP011108.1-5	GTTTCCATACCTCTAAGGAATTATTGAAACC	162	31	3	2	-4.80	80°C	35.48
	NZ_CP011108.1-6	GTTTCAATACTTCCTTAGAGGTATGGAAAC	563	30	9	8	-10.30	80°C	36.67
	NZ_CP011108.1-7	GTTTCCATACCTCTAAGGAAGTATTGAAAC	227	30	4	3	-7.40	80°C	36.67
	NZ_CP011108.1-8	GTTTCCATACCTCTAAGGAATTATTGAAAC	892	30	14	13	-4.00	80°C	33.33
Thermotoga maritima MSB8	NZ_CP011107.1-1	GTTTCAATAATTCCTTAGAGGTATGGAAAC	2694	30	41	40	-4.10	80°C	33.33
	NZ_CP011107.1-2	GTTTCAATAATTCCTTAGAGGTATGGAAAC	562	30	9	8	-4.10	80°C	33.33
	NZ_CP011107.1-3	GTTTCAATAATTCCTTAGAGGTATGGAAAC	566	30	9	8	-4.10	80°C	33.33
	NZ_CP011107.1-4	GTTTCCATACCTCTAAGGAATTATTGAAAC	1629	30	25	24	-4.00	80°C	33.33
	NZ_CP011107.1-5	GTTTCCATACCTCTAAGGAATTATTGAAACC	162	31	3	2	-4.80	80°C	35.48
	NZ_CP011107.1-6	GTTTCAATACTTCCTTAGAGGTATGGAAAC	563	30	9	8	-10.30	80°C	36.67
	NZ_CP011107.1-7	GTTTCCATACCTCTAAGGAAGTATTGAAAC	227	30	4	3	-7.40	80°C	36.67
	NZ_CP011107.1-8	GTTTCCATACCTCTAAGGAATTATTGAAAC	892	30	14	13	-4.00	80°C	33.33
Thermotoga naphthophila RKU-10	NC_013642.1-1	GTTTCAATAGTTCCTTAGAGGTATGGAAAC	1430	30	22	21	-6.70	80°C	36.67
	NC_013642.1-2	GTTTCCATACCTCTAAGGAACTATTGAAAC	299	30	3	2	-4.00	80°C	36.67

	NC_013642.1-3	GTTTCAATAATTCCTTAGAGGTATGGAAAC	686	30	11	10	-4.10	80°C	33.33
	NC_013642.1-4	GTTTCAATAGTTCCTTAGAGGTATGGAAAC	1219	30	19	18	-6.70	80°C	36.67
	NC_013642.1-5	GTTTTTATCTTCCTAAGAGGAATATGAAC	1817	28	28	27	-5.40	80°C	31.03
	NC_013642.1-6	GTTTCAATAATTCCTTAGAGGTATGGAAAC	891	30	14	13	-4.10	80°C	33.33
	NC_013642.1-7	GTTTCCATACCTCTAAGGAATTATTGAAAC	430	30	7	6	-4.00	80°C	33.33
	NC_013642.1-8	GTTTCAATAATTCCTTAGAGGTATGGAAAC	1431	30	22	21	-4.10	80°C	33.33
Thermotoga sp. RQ2	NC_010483.1-1	GTTTCAATAGTTCCTTAGAGGTATGGAAAC	1022	30	16	15	-6.70	80°C	36.67
	NC_010483.1-2	GTTTCCATACCTCTAAGGAACTATTGAAAC	1640	30	25	24	-4.00	80°C	36.67
	NC_010483.1-3	GTTTCCATACCTCTAAGGAACTATTGAAAC	429	30	7	6	-4.00	80°C	36.67
	NC_010483.1-4	GTTTCCATACCTCTAAGGAATTATTGAAAC	1833	30	28	27	-4.00	80°C	33.33
	NC_010483.1-5	GTTTCAATAGTTCCTTAGAGGTATGGAAAC	2091	30	32	31	-6.70	80°C	36.67
	NC_010483.1-6	GTTTCAATACTTCCTTAGAGGTATGGAAAC	1029	30	16	15	-10.30	80°C	36.67
	NC_010483.1-7	GTTTCCATACCTCTAAGGAATTATTGAAAC	556	30	9	8	-4.00	80°C	33.33
	NC_010483.1-8	GTTTCAATAATTCCTTAGAGGTATGGAAAC	230	30	4	3	-4.10	80°C	33.33
Thermotoga sp. RQ7	NZ_CP007633.1-1	GTTTCAATCCTTCCTTAGAGGTATGGAAACA	165	31	3	2	-8.00	80°C	38.71
	NZ_CP007633.1-2	GTTTCCATACCTCTAAGGAAGTATTGAAAC	427	30	7	6	-7.40	80°C	36.67
	NZ_CP007633.1-3	GGTTTCAATACTTCCTTTGAGGTATGGAAAC	231	31	4	3	-10.10	80°C	38.71
	NZ_CP007633.1-4	GTTTTTATCTTCCTAAGAGGAATATGAAC	3359	29	52	51	-5.40	80°C	31.03
	NZ_CP007633.1-5	GTTTCAATACTTCCTTTGAGGTATGGAAAC	691	30	11	10	-10.10	80°C	36.67
	NZ_CP007633.1-6	GTTTCAATACTTCCTTAGAGGTATGGAAAC	369	30	6	5	-10.30	80°C	36.67
	NZ_CP007633.1-7	GTTTCCATACCTCTAAGGAAGTATTGAAAC	229	30	4	3	-7.40	80°C	36.67
	NZ_CP007633.1-8	GTTTCAATACTTCCTTTGAGGTATGGAAAC	698	30	11	10	-10.10	80°C	36.67
Thermotoga thermarum DSM 5069	NC_015707.1-1	GTTTGAACCCTACCTATAAGGAATGGAAAC	1852	30	28	27	-2.00	80°C	40.00
	NC_015707.1-2	GTTTGGACACTACCTATGAGGAATGGAAAC	1444	30	22	21	-1.50	80°C	43.33
	NC_015707.1-3	GTTTGGACACTACCTATGAGGAATGGAAAC	1215	30	19	18	-1.50	80°C	43.33

	NC_015707.1-4	GTTTGAACCCTACCTATAAGGAATGGAAAC	1964	30	30	29	-2.00	80°C	40.00
	NC_015707.1-5	GTTTGGACACTACCTATGAGGAATGGAAAC	1968	30	30	29	-1.50	80°C	43.33
	NC_015707.1-6	GTTTCAATCGAACTTAAGAGGGATGGAAAC	2623	30	40	39	-4.40	80°C	40.00
Thermodesulfobacterium geofontis OPF15	NC_015682.1-1	GGAGATGAATGCAAGGTTTGAGGCT	229	25	5	4	0.00	83°C	48.00
	NC_015682.1-2	GTTGAAACCTGCCCTGGATTAAAAGGGATTGCGAA	3107	35	42	41	-6.00	83°C	45.71
	NC_015682.1-3	GTTTCAATCCACCAAAGAGGAATTTAAAC	3557	29	55	54	-2.00	83°C	34.48
Thermocrinis ruber strain DSM 23557	NZ_CP007028.1-1	CTAAAACTGAAGAAGTGTTAGAGGA	94	25	2	1	-3.60	85°C	36.00
	NZ_CP007028.1-2	CTTTCAACTCCACACGGTACATTAGGAAC	1240	29	19	18	-0.90	85°C	44.83
	NZ_CP007028.1-3	CTTTCAACTCCACACGGTACATTAGGAAC	700	29	11	10	-0.90	85°C	44.83
	NZ_CP007028.1-4	GTTCCTAATGTACTGTGTGGAGTTGAAAG	1163	29	18	17	-2.30	85°C	41.38
	NZ_CP007028.1-5	GTTCCTAATGTACCGTGTGGAGTTGAAAG	907	29	14	13	-3.70	85°C	44.83
	NZ_CP007028.1-6	GTTTCAATCCCCTATAGGCACTT	688	23	11	10	0.00	85°C	43.48
	NZ_CP007028.1-7	GTTTATTAAATGCCTATAGGGGATTGAAAC	165	30	3	2	-3.50	85°C	33.33
	NZ_CP007028.1-8	GGAGGAGTCGAACCTCCGACCTC	98	23	2	1	-7.90	85°C	65.22
	NZ_CP007028.1-9	CTTTCAACTCCACACGGTACATTAGGAAC	560	29	9	8	-0.90	85°C	44.83
Thermotoga neapolitana DSM 4359	NC_011978.1-1	GTTTCCATACCTCAAAGGAAGTATTGAAAC	368	30	6	5	-6.80	87.5°C	36.67
	NC_011978.1-2	GTTTCAATACTTCCTTAGAGGTATGGAAAC	362	30	6	5	-10.30	87.5°C	36.67
	NC_011978.1-3	GTTTCCATACCTCAAAGGAAGTATTGAAAC	437	30	7	6	-6.80	87.5°C	36.67
	NC_011978.1-4	GTTTCAATACTTCCTTAGAGGTATGGAAAC	430	30	7	6	-10.30	87.5°C	36.67
	NC_011978.1-5	GTTTTTATCTTCCTAAGAGGAATATGAAC	1870	29	29	28	-5.40	87.5°C	31.03
	NC_011978.1-6	CGGGTTTCAATACTTCCTTAGAGGTATGGAAAC	168	33	3	2	-10.30	87.5°C	42.42
	NC_011978.1-7	GTTTCCATACCTCTAAGGAAGTATTGAAAC	230	30	4	3	-7.40	87.5°C	36.67
	NC_011978.1-8	GTTTCAATACTTCCTTTGAGGTATGGAAAC	361	30	6	5	-10.10	87.5°C	36.67
Thermofilum adornatus	NC_022093.1-1	CTTTCAATTCATTCTTTTCTGATAC	156	25	3	2	0.00	92°C	32.00
	NC_022093.1-2	ACCTTTCAATTATTTCTGAGTTGCATC	90	27	2	1	-0.20	92°C	33.33

	NC_022093.1-3	CTTTCAATTCATTCTTTTCTGATAC	279	25	5	4	0.00	92°C	32.00
	NC_022093.1-4	CTTTGAATTCTTTCTGAGTTGCATC	92	25	2	1	-2.70	92°C	36.00
	NC_022093.1-5	CTTTCAATTCATTCGTTTCTGATAC	224	25	4	3	0.00	92°C	32.00
	NC_022093.1-6	CTTTCAATTCATTCTTTTCTGATAC	154	25	3	2	0.00	92°C	32.00
	NC_022093.1-7	CTTTCAATTCATTCTTTTCTGATAC	215	25	4	3	0.00	92°C	32.00
	NC_022093.1-8	CTTTCAATTCTTTCTGAGTTGCATC	158	25	3	2	-4.30	92°C	36.00
	NC_022093.1-9	CTTTCAATTCTTTCTTAGTTGCATC	824	25	13	12	0.00	92°C	32.00
	NC_022093.1-10	CTTTCAATTCTTTCTTAGTTGCATC	413	25	7	6	0.00	92°C	32.00
	NC_022093.1-11	GATGCAACTGAGAAAGAATTGAAAGC	282	26	5	4	0.00	92°C	38.46
	NC_022093.1-12	CTTTCAATTCATTCGTTTCTGATAC	349	25	6	5	0.00	92°C	32.00
	NC_022093.1-13	CTTTCAATTCTTTCTTAGTTGCATC	872	25	14	13	0.00	92°C	32.00
	NC_022093.1-14	CTTTCAATTCATTCGTTTCTGATAC	689	25	11	10	0.00	92°C	32.00
	NC_022093.1-15	CCTTCAATTCTTTCTTAGTTGCATC	1761	25	27	26	0.00	92°C	36.00
	NC_022093.1-16	CCTTCAATTCTTTCTTAGTTGCATC	1139	25	18	17	0.00	92°C	36.00
	NC_022093.1-17	CTTTCAATTCTTTCTTAGTTGCATC	694	25	11	10	0.00	92°C	32.00
Thermus sp. CCB_US3_UF1	NC_017278.1-1	GTAGTCCCCACGCACGTGGGGATGGACC	210	28	4	3	-14.30	92.4°C	67.86
	NC_017278.1-2	GTTTCAAACCCTCATAGGTACGGTCAGAAC	1178	30	18	17	-2.00	92.4°C	46.67
	NC_017278.1-3	CTTTGAACCGTACCTATAAGGGTTTGAAAC	981	30	15	14	-5.90	92.4°C	40.00
	NC_017278.1-4	CTTTGAACCGTACCTATAAGGGTTTGAAAC	1577	30	24	23	-5.90	92.4°C	40.00
	NC_017278.1-5	GTTGCAAAAGTGGCTTCCCCGCAAGGGGATTGCGAC	1382	36	19	18	-14.60	92.4°C	58.33
	NC_017278.1-6	GTCGCAATCCCCTTACGGGGAAGCCACTTTTGCAAC	719	36	10	9	-10.70	92.4°C	55.56
	NC_017278.1-7	GTCGCAATCCCCTTACGGGGAAGCCACTTTTGCAAC	936	36	13	12	-10.70	92.4°C	55.56
	NC_017278.1-8	CGTAGTCCCCACACGCGTGGGGATGGACC	151	29	3	2	-14.60	92.4°C	68.97
