# Supplementary material for: Comparison of Structural Features of CRISPR-Cas Systems in Thermophilic Bacteria
Source: Microorganisms. 2023 Sep 10;11(9):2275. doi: 10.3390/microorganisms11092275 (PMC10536717; doi:10.3390/microorganisms11092275)
Supplement: Supplementary file 1 [file microorganisms-11-02275-s001.zip › Table S3.pdf]

Table S3. The analysis results of 209 different repeat sequences and CRISPRmap database.

DR sequence	**Structures**	**Sequences**	**Superclass**
ACCTTTCAATTATTTCTGAGTTGCATC	-	-	D
ACTATTTTCAGGATAGGTAGGCTAAAAAC	-	-	-
AGGTTTGTTGCTTCATGTGTGTAAGAAAA	-	-	-
AGTAGTCCCCACGCACGTGGGGATGGCCCG	-	-	B
AGTCCGAAAGAAGGTCCTCTCTGAAAGAGATGG	motif 15	-	-
AGTTTTTACGATACCTATGAGGAATTGAAACA	-	family 2	A
ATCAATCAATTACTTATACATCTAA	-	-	D
ATCATCTGACTACCTGACTACCG	-	-	D
ATGGCCCGGAAACGCGCGGGGCG	-	-	D
ATTGTCCCCACACGCGTGGGGGTGTACACCG	-	-	B
ATTTACATCCCTCATAGTTCAGATAAAAC	motif 13	family 2	A
ATTTCAATTCCTACAAGGTAAGGTACAAAC	-	family 2	A
ATTTCAATTCCTCCAAGGTAAGGTAAAAAC	-	family 2	A
ATTTCAATTCCTGCAAGGTAAGGTACAAAC	-	family 2	A
ATTTCTATTCCTCATAGGTAGATTCTAAAC	-	family 2	A
CAACTCCACACGGTACATTAGAAAC	-	family 6	D
CACTATTTTCAGGATAGGTAGGCTAAAAAC	-	family 2	B
CAGTTCTAGCAGTTAGAACGAGTTGTAAACCT	-	-	C
CCAGGGCCGCCTGGGCGAGGAGGA	-	-	D
CCATCTCCGCCTTGAGCTCCTGGCGCA	-	-	D
CCCTTTTCACCATAAAATCAATACTTTTCAAC	-	-	-
CCGTTCCCCGCGGGCCGGAAGGGGTG	-	-	-
CCTTCAATTCTTTCTTAGTTGCATC	-	family 3	D
CGACATCCCGCGCTGACCGGGGGCCACGG	-	-	-
CGACCGGCGGCCCGCTCACGGCC	-	-	D
CGCCGTCGTTTTTATAAGGGGAATAGAAACCC	-	-	-
CGCGGGCCCCGCGCATGCGGGACGCGGGC	-	-	-
CGGACCACCCCCGCCTGCGCGGGGAGCAC	motif 1	family 1	B
CGGCCCCGCATCGCCCGGGCGCCGC	-	-	D
CGGCGGCGCGCGGACACGACGGGTGA	-	-	-
CGGGCCATCCCCACGCGTGTGGGGACTAC	motif 1	family 1	B
CGGGTTTCAATACTTCCTTAGAGGTATGGAAAC	-	family 2	-
CGGTCCATCCCCACGGGCGTGGGGACTAC	motif 1	family 1	B
CGGTTCACCCCCACGCGTGTGGGGACAAT	motif 1	family 1	B
CGTAGTCCCCACACGCGTGGGGATGGACC	-	family 1	B
CGTGCAGGCCTCGTATCTGCTCTGAGGTGCAAG	-	-	A
CGTTCACCACCAGCAGGGCCGCC	-	-	D
CTAAAACTGAAGAAGTGTTAGAGGA	-	-	D
CTATAGTTGTTGGTTGTTGGTTTT	-	-	D
CTCCCGTCCCCGGGCCCAGCGGGC	-	-	D
CTCGCAGTGACAAATGTGATCAGGG	-	-	D

CTGCTCCCCGCGCACGCGGGGATGGTCCC	motif 1	family 1	B
CTGTAACGCGGATACCGCGTTACA	motif 11	-	D
CTTCTAAACCTACATAGGATATTTCAAAC	-	family 12	A
CTTTATATCCCACATGGTTCAGATGAAAC	-	family 2	A
CTTTATATCTCACATGGTTCAGATGAAAC	-	-	A
CTTTCAACTCCACACGGTACATTAGAAAC	-	family 2	A
CTTTCAACTCCACACGGTACATTAGGAAC	-	-	-
CTTTCAATTCATTCGTTTCTGATAC	-	family 3	D
CTTTCAATTCATTCTTTTCTGATAC	-	family 3	D
CTTTCAATTCCAGTATGGTTGGATTAAATC	-	-	A
CTTTCAATTCCGTTCTACGGAATTTGGTCTTGAGGC	-	-	C
CTTTCAATTCCTTATAGGTAGGCTAAAAAC	-	family 2	A
CTTTCAATTCTTCTAAAGTCTTATTGGAAC	-	family 4	A
CTTTCAATTCTTTCTGAGTTGCATC	-	family 3	D
CTTTCAATTCTTTCTTAGTTGCATC	-	family 3	D
CTTTCAATTCTTTTAAAGTCTTATTGGAAC	-	family 4	A
CTTTGAACCGTACCTATAAGGGTTTGAAAC	-	family 2	A
CTTTGAATTCTTTCTGAGTTGCATC	motif 11	-	D
CTTTTGACCGTACCTATGAGGGTTTGAAAC	-	-	A
GAGGTCGCCGGTGTTCATCGCCATC	-	-	D
GATGCAACTGAGAAAGAATTGAAAGC	-	family 3	D
GATTCAATCGAACCGATACGGAATGGAAAC	-	family 2	A
GATTTAAGCGGGCGCATCCGTTTTGCGGA	-	-	-
GCCACCGGGCCTCAGCGCCACCGTC	motif 11	-	-
GCGAGGGAGGCTGCAGCGTGCAT	-	-	D
GCGCCCCAGGAGCACGGTGCGCGG	-	-	D
GCGGAGCTTCGGGCCCCGTGGGA	-	-	D
GCGGCGGCAGGCGCGTGAGGGGCCGCG	-	-	-
GCGGGACCCCGGACGGGCGGGCGG	-	-	D
GCGTTCTCCTGGCCCGGCAGGGCCGG	-	-	-
GGAGATGAATGCAAGGTTTGAGGCT	-	-	D
GGAGGAGTCGAACCTCCGACCTC	-	-	D
GGATCATCCCCGCATGCGCGGGGAGCAC	motif 7	family 1	B
GGGACCATCCCCGCGTGCGCGGGGAGCAG	motif 1	family 1	B
GGGATCATCCCCGCGTGCGCGGGGAGCAC	motif 1	family 1	B
GGGATCATCCCCGCGTGCGCGGGGAGCAG	motif 1	family 1	B
GGGCCATCCCCACGTGTGTGGGGACT	-	-	D
GGTTGCGATCCCTCTGAGGGGTGATGAGGGC	-	-	B
GGTTTCAATACTTCCTTTGAGGTATGGAAAC	-	-	-
GGTTTTTTCAATTCGAATAATCGAAAGAAC	-	-	-
GTAGAAACCTGCCCTACTTCAAAAGGGATTGCGAC	motif 16	-	E
GTAGGAAACAAGACCTCATTTAAAAGGGATTGCGAC	-	family 11	E
GTAGTCCCCACACGCGTGGGGATGGACCG	motif 1	family 1	B

GTAGTCCCCACGCACGTGGGGATGGACC	motif 7	family 1	B
GTAGTCCCCACGCATGTGGGGATGGCCCG	motif 1	family 1	B
GTCAGTGCGGGCGGGGGAGCAGG	-	-	D
GTCCGGGAGGCGCGGATGAGCGCC	motif 11	-	D
GTCCTCATCACCCCTCAGAGGGATCGCAAC	-	-	B
GTCCTCATCACCCCTCGGAGGGATCGCAAC	-	-	B
GTCGCAATCCCCTGACGGGGAAGCATCTCGTGCAAC	motif 4	family 8	E
GTCGCAATCCCCTTACGGGGAAGCCACTTTTGCAAC	motif 4	family 8	E
GTCGCAATCCCCTTACGGGGCTAAGTGG	-	-	-
GTCGCAATCCCCTTACGGGGCTAAGTGGTTTGCAAC	-	-	C
GTCGCAATCCCCTTACGGGGCTCAATCCCTTGCAAC	motif 4	family 8	E
GTCGCAATCCCGTCTACTTTTTCGGGCATTTCAAC	-	-	F
GTCGCAATCCCTTATTCTTCAGGGAATTTTTCTAAC	-	family 11	E
GTCGCCCGCTGTGCGCGGGCGTGGGTTGAAAC	motif 3	family 5	C
GTCGCTCATCACCCCTGGAGGGATCGCAAC	-	-	B
GTGAGAAAACCTTGCCTGATTAAGAAGGCATTACGAC	-	family 11	E
GTGCTCAACGCCTTACGGCATCAGAGGTAGAGGCAC	motif 15	-	F
GTGCTCCCCGCGCACGCGGGGATGATCCC	motif 1	family 1	B
GTGCTCCCCGCGCAGGCGGGGGTGATCCG	motif 1	family 1	B
GTTAAAAAACCTAATTCCATAAATGGAATTCAAAC	-	-	E
GTTACTAGCTTACCTATGAGGGGTTGAAAC	-	family 2	A
GTTACTAGCTTACCTATGAGGGGTTGAAACAT	-	family 2	-
GTTCACAGCCTAACTAAAAGGAATGGAAAC	-	family 2	A
GTTCATATTCCTCTTAGGAAGATAAAAAC	-	family 2	A
GTTCCAATAAGACTTTAAAAGAATTGAAAG	-	family 4	A
GTTCCTAATGTACCGTGTGGAGTTGAAAG	-	-	A
GTTCCTAATGTACTGTGTGGAGTTGAAAG	-	-	A
GTTCTACTGTAACTTAGAAGTTTTTTGTG	-	-	-
GTTCTTAGTCTACCTATAAGGGATTGAAAC	-	family 2	A
GTTGAAAAGTATTGATATTATGTCGAGAAGG	-	-	-
GTTGAAACCTGCCCTGGATTAAAAGGGATTGCGAA	-	family 11	E
GTTGAAATGCCCGAAAAAATCGGCGGGATTGCGAC	-	-	F
GTTGAAATGCCCGAAAAAGTAGACGGGATTGCGAC	-	-	F
GTTGCAAAAGTGGCTTCCCCGCAAGGGGATTGCGAC	motif 4	family 8	E
GTTGCAAGGGATTGAGCCCCGTAAGGGGATTGCGAC	motif 4	family 8	E
GTTGCACGAGATGCTTCCCCGTCAGGGGATTGCGACC	motif 4	family 8	E
GTTGCGATCCCTCCAGGGGTGATGAGCGAC	-	-	B
GTTGCGATCCCTCTAGGGGTGATGAGCGGAC	-	-	B
GTTGCGATCCCTCTGAGGGGTGATGAGGAC	-	-	B
GTTGCGATCCCTCTGAGGGGTGATGAGGACC	-	-	B
GTTGGAAAACAAGACCTCATTTAAAAGGGATT	-	family 11	E
GTTTAAATCCCACTTGTTCAAATAAAAC	-	family 2	A
GTTTAAATCCCACTTGTTCAGATAAAAC	-	family 2	A

GTTTAGAACATACCTATGAGGAATGGAAAC	-	-	A
GTTTAGAATCTACCTATGAGGAATGAAAAC	-	family 2	A
GTTTAGAATCTACCTATGAGGAATGGAAAC	-	family 2	A
GTTTATAACCCACAATGGTTCTACCTAAAC	-	family 12	A
GTTTATTAAATGCCTATAGGGGATTGAAAC	-	-	A
GTTTCAAACCCTCATAGGTACGGTCAGAAC	-	family 2	A
GTTTCAATAATTCCTTAGAGGTATGGAAAC	-	family 2	A
GTTTCAATACTTCCTTAGAGGTATGGAAAC	-	family 2	A
GTTTCAATACTTCCTTTGAGGTATGGAAAC	-	family 2	A
GTTTCAATAGTTCCTTAGAGGTATGGAAAC	-	family 2	A
GTTTCAATCCACCAAAGAGGAATTTAAAC	-	family 2	A
GTTTCAATCCCCTATAGGCACTT	-	-	D
GTTTCAATCCCTAATAGGTATGCTAAAAAC	-	family 2	A
GTTTCAATCCCTCTTAGGTAGGCTAAAAAC	-	family 2	A
GTTTCAATCCCTTATAGGTAAGCTAAAAAC	-	family 2	A
GTTTCAATCCTTCCTTAGAGGTATGGAAACA	-	-	-
GTTTCAATCGAACCTTAGAGGGATGGAAAC	-	family 2	A
GTTTCAATCGAACTTAAGAGGGATGGAAAC	-	family 2	A
GTTTCAATTCCACTATGGTTAGATTAAATC	-	family 2	A
GTTTCAATTCCTACAAGGTAAGGTACAAAC	-	family 2	A
GTTTCAATTCCTCATAGGTACGATCAAAAC	-	family 2	A
GTTTCAATTCCTCATAGGTAGGCTAAAAAC	-	family 2	A
GTTTCAATTCCTGATAGGTAGGCTAAAAAC	-	family 2	A
GTTTCAATTCCTTATAGGTAGGCTAAAAAC	-	family 2	A
GTTTCCATACCTCAAAGGAAGTATTGAAAC	-	family 2	A
GTTTCCATACCTCTAAGGAACTATTGAAAC	-	family 2	A
GTTTCCATACCTCTAAGGAAGTATTGAAAC	-	family 2	A
GTTTCCATACCTCTAAGGAATTATTGAAAC 14	-	family 2	A
GTTTCCATACCTCTAAGGAATTATTGAAACC	-	-	A
GTTTCCATCCCTCATAGGACCTCTCTTAAAC	-	-	-
GTTTCCATCCCTCATAGGAGCCTTCTAAAC	-	-	-
GTTTCCATCCCTCTAAGGTTCGATTGAAAC	-	family 2	A
GTTTCCATCTCTTTCAGAGAGAACCTTCTTTCGGAC	motif 15	-	F
GTTTCCATTCCTCATAGATTCGATTGAAC	-	-	A
GTTTCCATTCCTCATAGGTAGATTCTAAAC	-	family 2	A
GTTTCCATTCCTCGTAGGTAGGCTGGGAAC	-	-	A
GTTTCCATTCCTCTTAGATTCGATTGAAAC	-	-	A
GTTTCGACAGTACCTATGAGGGCTTGAAAC	-	family 2	A
GTTTCTAATGTACCGTGTGGAGTTGAAAG	-	family 2	A
GTTTCTACCTTACCTTGGAGGAATTGAAAC	-	family 2	A
GTTTGAAATGCCCTATAAGGGATTGTGAC	-	family 2	A
GTTTGAACCCTACCTATAAGGAATGGAAAC	-	family 2	A
GTTTGATCTGAACTATGTGGGATGTGAAC	-	-	A

GTTTGGACACTACCTATGAGGAATGGAAAC	-	family 2	A
GTTTTAGACCTTCCTATAAGGGATGGAAAC	-	family 2	A
GTTTTATCTGAACAAGTGGGATTTAAAC	-	family 2	A
GTTTTATTTGAACAAGTGGGATTTAAAC	-	family 2	A
GTTTTCATTCCTCATAGGTAGATTCTAAAC	-	family 2	A
GTTTTGAGCCTACCTACAAGGAATTGAAAC	-	-	A
GTTTTGAGCCTACCTATGAGGAATTGAAAC	-	family 2	A
GTTTTGCAGCCAGTAATTGAGAGACTGAG	-	-	B
GTTTTTACGATACCTATGAGGAATTGAAAC	-	family 2	A
GTTTTTAGCCTACCTAAAAGGGATTGAAAC	-	family 2	A
GTTTTTAGCCTACCTATAAGGAATTGAAAC	-	family 2	A
GTTTTTAGCCTACCTATAAGGGATTGAAAC	-	family 2	A
GTTTTTAGCCTACCTATGAGGAATTGAAAC	-	family 2	A
GTTTTTAGCTTACCTATAAGGGATTGAAAC	-	family 2	A
GTTTTTAGCTTACCTATGAGGGATTGAAAC	-	family 2	A
GTTTTTAGTCTACCTATGAGGGATTGAAAC	-	family 2	A
GTTTTTATCTTCCTAAGAGGAATATGAAC	-	family 2	A
TAAAAGAAGCGGGTTTCCCACTTCTTTTAG	-	-	-
TATCTCTCCTACTATCTTCTTTGT	-	-	-
TCAACCCATATAGTAATTTAAAA	-	-	D
TCGCAATCCCTTTTAAATGAGGTCTTGTTTTCCAAC	-	family 11	E
TCTCAGCGGGCCGCCCGCCTCAG	-	-	D
TCTGCGGTGGTTGCGGTGGTTTGCGGTGGTTGTG	-	-	-
TGAGACTGCTTCGCTGCGCTCGCAGTGACAGG	-	-	-
TGATTCCTGTACTGATTATAGTTCGA	motif 11	-	-
TTATTTTTATTTTTGTATTACATCT	-	-	-
TTCATCACGCAAGGCACCTAGGGA	-	-	D
TTTTTAGCCTATCTATGAGGGATTGAAAC	-	family 2	A
CGGGTGCGGGGCGCCCTGTTGGCCCGTCC	-	-	-
CGGTCCATCCCCACGTGCGTGGGGCTCAC	motif 1	family 1	B
GACCAGGCCACCATCAACCTCGG	-	-	D
GAGAGCCCCACGCACGTGGGGATGGACCG	motif 1	family 1	B
GGACCATCCCCGCATGCGCGGGAAGCAC	motif 7	family 1	B
GGGACCATCCCCGCATGCGCGGGGAGCAC	motif 1	family 1	B
GGTCCATCCCCACGTGCGTGGGGAGCAT	motif 7	family 1	B
GGTCCATCCCCGCATGCGCGGGGAGCAC	-	-	D
GTGCTCCCCACGCACGTGGGGATGGTCCG	motif 1	family 1	B
GTGCTCCCCGCGCATGCGGGGATGGTCC	motif 7	family 1	B
GTTTCCATTCCTCATAGGTATGTTCTAAAC	-	-	-
